# The contribution of stimulating multiple body parts simultaneously to the illusion of owning an entire artificial body

**DOI:** 10.1371/journal.pone.0233243

**Published:** 2021-01-25

**Authors:** Sophie H. O’Kane, H. Henrik Ehrsson

**Affiliations:** Department of Neuroscience, Karolinska Institutet, Stockholm, Sweden; Anglia Ruskin University, UNITED KINGDOM

## Abstract

The full-body ownership illusion exploits multisensory perception to induce a feeling of ownership of an entire artificial body. Although previous research has shown that synchronous visuotactile stimulation of a single body part is sufficient for illusory ownership of the whole body, the effect of combining multisensory stimulation across multiple body parts remains unknown. Therefore, 48 healthy adults participated in a full-body ownership illusion with conditions involving synchronous (illusion) or asynchronous (control) visuotactile stimulation to one, two, or three body parts simultaneously (2×3 design). We used questionnaires to isolate illusory ownership of five specific body parts (left arm, right arm, trunk, left leg, right leg) from the full-body ownership experience and sought to test not only for increased ownership in synchronous versus asynchronous conditions but also for potentially varying degrees of full-body ownership illusion intensity related to the number of body parts stimulated. Illusory full-body ownership and all five body-part ownership ratings were significantly higher following synchronous stimulation than asynchronous stimulation (*p*-values < .01). Since non-stimulated body parts also received significantly increased ownership ratings following synchronous stimulation, the results are consistent with an illusion that engages the entire body. Furthermore, we noted that ownership ratings for right body parts (which were often but not always stimulated in this experiment) were significantly higher than ownership ratings for left body parts (which were never stimulated). Regarding the effect of stimulating multiple body parts simultaneously on explicit full-body ownership ratings, there was no evidence of a significant main effect of the number of stimulations (*p* = .850) or any significant interaction with stimulation synchronicity (*p* = .160), as assessed by linear mixed modelling. Instead, median ratings indicated a moderate affirmation (+1) of an illusory full-body sensation in all three synchronous conditions, a finding mirrored by comparable full-body illusion onset times. In sum, illusory full-body ownership appears to be an ‘all-or-nothing’ phenomenon and depends upon the synchronicity of visuotactile stimulation, irrespective of the number of stimulated body parts.

## Introduction

How does one come to perceive that one’s body, the single, integrated biological entity in which one senses and acts upon one’s environment, belongs exclusively to oneself? What are the neurocognitive principles governing the perception of one’s own body not as a set of fragmented body parts but as the gestalt that delineates the boundaries between what is the self and what is not? The feeling of ‘body ownership’ [1–4] attracts attention across diverse academic fields, although the distinction between part and whole, herein referred to as ‘body-part ownership’ and ‘full-body ownership’, respectively [2,3,5–9], has been studied less often. In cognitive neuroscience, the discovery of the rubber hand illusion [10] led to an exciting expansion of empirical research towards understanding the perceptual processes and neural mechanisms that underpin ownership of a single limb in healthy individuals [11,12]. Using experimental conditions to exploit the basic principles of multisensory integration [13], this simple perceptual illusion provides an exquisite demonstration of the malleability of the sense of body ownership amongst healthy people [2,3]. However, in addition to inducing a sense of ownership of a prosthetic hand, [7] revealed that the illusory experience of ownership could be extended to encompass an entire artificial body, which opened the way for experimental research on full-body ownership alongside body-part ownership.

During the ‘full-body ownership illusion’, also referred to as the ‘body-swap illusion’ [7], tactile stimulation is administered to the participant’s real body in spatio-temporal synchrony with visual feedback of identical stimuli being applied to a plastic mannequin, which is presented through a head-mounted display from the natural (first-person) viewpoint. Subjective reports of referral of touch (the illusory experience of directly feeling the touches applied to the artificial body) plus some degree of illusory ownership of the entire mannequin are well supported in the majority of participants [5–7,9]. These results support multisensory integration of visual, tactile and proprioceptive input as an essential framework to investigate the feeling of full-body ownership [2,3,14,15].

In order for the illusory percept of ownership to arise, multisensory stimulation must obey basic principles that enable successful integration. Visuotactile stimulation must be temporally synchronous, while temporally asynchronous visuotactile stimulation provides a reliable control condition for most studies [5–7,16–18]. Moreover, visuotactile stimulation must be spatially congruent, i.e., applied to the corresponding body parts and in the same direction, and the shape and structure of the artificial body in view must match the shape and structure of a human body, as the illusion cannot be elicited by a block of wood [3,7]. The size [19,20] and gender [7] of the humanoid body seem to be less important, and the illusion also works well using the bodies of human strangers [16,21,22] and computer-generated bodies in virtual reality [23,24]. For the paradigm used in the current study, a humanlike body is presented in an anatomically congruent position from the first-person perspective [5–7,16]. Although illusory ownership can be induced over a false body presented in a mirror [17], the illusion is generally less effective when the false body is presented from a third-person perspective [8,9,25,26]. The above rules correspond very well to (i) the temporal and spatial principles of multisensory integration, (ii) prior information influencing multisensory causal inference, and (iii) cross-modal processing in body-centred coordinates in peripersonal space, which collectively suggest that participants experience a perceptual illusion of the mannequin’s body as their own body during the illusion (Ehrsson 2020).

To date, few studies have explicitly examined how the full-body ownership percept is established during the full-body ownership illusion and how such a whole-body gestalt relates to the sense of ownership of specific body parts. Previous research has demonstrated comparable magnitudes of subjective ownership of a mannequin’s body irrespective of which singular body part receives illusion-inducing synchronous visuotactile stimulation [5–7]. In [5], visuotactile stimulation was applied to the right hand, the abdomen, or the right leg, and ownership of each of these three body parts was assessed. The authors observed increased ownership applying not only to the specific body part receiving synchronous visuotactile stimulation but also to the other (two) non-stimulated body parts, suggesting that the illusion of ownership had “spread” to encompass the whole body, although explicit sensations of full-body ownership were not examined in this study. In addition to questionnaire data, [7] applied visuotactile stimulation to either the right hand or the abdomen and used threat-evoked skin conductance responses (SCRs) (μS) towards a knife, always aimed at the mannequin’s abdomen, for an objective quantification of illusory ownership [27–29]. Critically, the magnitude of participants’ SCRs (μS) was not affected by whether the stimulated body part was the same one subsequently threatened with the knife (the abdomen) or not (the hand) [7]. These behavioural insights support the hypothesis that the feeling of ownership during the perceptual illusion is not = restricted to the body part receiving synchronous visuotactile stimulation but instead becomes generalised into a global percept of ownership corresponding to the entire body plan. However, the mechanisms of this “spread of ownership” from the synchronously stimulated body part to the rest of the body in a seamless percept of illusory full-body ownership remain to be fully understood. Specifically, more research is needed to better understand the relationship between body-part and full-body ownership. Is the whole simply the sum of the parts, or is the whole-body ownership experience a more complex, holistic percept that cannot be deduced entirely from its parts?

In a continuation from previous studies, which stimulated only a single body part at any given time, the present study set out primarily to examine whether stimulating multiple body parts simultaneously could manipulate the illusory feeling of full-body ownership into gradations of intensity related to the number of body parts stimulated (one, two or three). For example, during a related paradigm, the invisible full-body illusion [30], stimulating all invisible contours of the body plan, albeit sequentially, was beneficial in constructing the illusory percept of owning the entire invisible body. Likewise, perhaps when the false body is in full view, as is the case for experiments with a mannequin or a stranger’s body, the volume of multisensory information congruent with the illusory ownership percept might influence feelings of full-body ownership. Moreover, by supplying multiple stimulations simultaneously, these signals may be integrated within the same temporal binding window; the temporal properties of stimuli are known to influence the perception of multisensory illusions [31–35]. Unlike delivering visuotactile stimulations to multiple body parts sequentially, which can be performed to elicit a full-body ownership illusion [29], simultaneously activating representations for multiple body parts might co-activate more subsystems of neural populations that integrate visual, tactile, and proprioceptive signals across multiple body segments and the whole body [5,6]. Given the generally close relationship between temporal binding and multisensory integration and awareness [36], it is plausible that converging multisensory stimulation across multiple body parts would facilitate the illusion by increasing the amount of available perceptual evidence in support of the whole body being one’s own [8,15,37,38].

However, it is also possible that a maximal illusion is elicited by the synchronous visuotactile stimulation of one body part, as earlier studies have described the successful induction of a full-body ownership illusion by stimulating single body parts [5–7,16,17,19,39]. The lack of an additional effect from stimulating additional body parts would be no less interesting, as it would suggest that perceived full-body ownership is not simply constructed by summation of ownership across constituent body parts. In that case, the multisensory perception of illusory full-body ownership might be best conceived of as an ‘all-or-nothing’ phenomenon [40], rather than being graded by the number of stimulated body parts. After observing similar magnitudes of illusory ownership during the rubber hand illusion irrespective of which sensory modalities were used to induce it, [41] speculated that there might be an ‘all-or-nothingness’ to the sense of illusory ownership. This view suggests that the experience of illusory ownership, whether it applies to a rubber hand during the rubber hand illusion or a whole body during the full-body ownership illusion, may be conceived of as a binary perceptual decision driven by sensory causal inference. In such a model, sensory evidence is accumulated until causal inference links such sensations to their most probable cause [37]. Although the causal inference model has been used primarily to explain the rubber hand illusion [37,42] it is arguably valid for the experience of a full-body ownership illusion as well [15]. Therefore, it is plausible that the synchronous stimulation of a single body part during the full-body ownership illusion supplies sufficient sensory evidence for the causal inference of owning the whole body. The accumulation of further evidence by stimulating multiple body parts may simply be immaterial since the perceptual decision for illusory full-body ownership would have already been achieved by the stimulation of a single body part.

In light of these unanswered questions, the present study aimed to extend previous findings by first determining whether illusory full-body ownership could be potentiated by increasing the number of stimulations across multiple body parts simultaneously compared to the stimulation of fewer body parts or, indeed, even a single body part. To address this issue, we developed a questionnaire statement that explicitly described the sense of ownership of the mannequin’s *whole* body, in addition to statements about specific body parts (see below). Second, we aimed to detail the “spread of ownership” across the body plan, focusing on illusory body-part ownership and how this may change with respect to the number of stimulated body parts. We expected greater ownership for the body parts that we stimulated synchronously compared to asynchronously. However, we also had the novel question of whether illusory ownership for the non-stimulated body parts (i.e., the “spread of ownership”) would increase with an increasing number of stimulated body parts. Third, we were interested in examining whether body-part ownership—as it applies to both stimulated and non-stimulated body parts—is correlated with the subjective experience of full-body ownership. Finally, as we used questionnaire statements referring to illusory ownership for five different body parts (right arm, right leg, trunk, left arm, left leg), we had an unprecedented opportunity to investigate whether there would be differences in illusory body-part ownership for the right- versus left-sided body parts, in line with possible lateralisations between the left and right halves of the body.

To test these questions, we applied the full-body ownership illusion [7] and a 2×3 within-subjects design to examine the effects of synchronous (illusion) versus asynchronous (control) visuotactile stimulation involving one, two or three body parts simultaneously. For continuity with previous studies [5–7], the stimulated body parts consisted of (1) the trunk, (2) the trunk and the right arm, or (3) the trunk, the right arm and the right leg. The subjective questionnaire, probing illusory body-part and full-body ownership, was complemented by both threat-evoked skin conductance response (SCR) (μS) and illusion onset time (seconds) data in the same participants; measures intended to probe the physiological and temporal dimensions of the full-body ownership illusion, respectively [7]. As far as we are aware, the present study is the first to explicitly measure illusion onset time using a specifically designed statement to capture full-body ownership beyond the ownership of body parts for a visuotactile full-body ownership illusion (for a visuomotor full-body illusion, see [43]). Previous studies have used wordings such as “please indicate when it feels like the mannequin (or avatar) is your body” and concluded that the onset is rather fast, occurring in approximately the first 10–12 seconds [6,21]. However, these studies did not emphasise the onset of illusory ownership sensations for the whole body, as in the present study.

## Methods

### Participants

Forty-eight healthy adults, a pre-determined sample size based upon previous behavioural studies using a full-body ownership illusion [7,22] (23; N = 40, 8; N = 32) and a counterbalanced experimental design (see ‘Methods –Procedures’ for details), were recruited to participate in the experiment via online advertisements, posters and personal communication; 28 males, 20 females, mean age 26.9 ± 6.2 years, age range: 19–43 years, 47 right-handed, 1 left-handed (self-reported). All had normal or corrected-to-normal vision and were instructed to wear comfortable clothing that would not interfere with the delivery of tactile stimulation, i.e., no buttoned shirts or high-waisted jeans, both of which impeded the delivery of the stimuli during the pilot. All recruits were naïve to the full-body ownership illusion, confirming that they had not participated in a similar study before based upon minimal information, such as viewing a mannequin’s body in a head-mounted display (HMD). Participants provided written informed consent, and the given information did not explicate the purposes of this specific experiment or the details of the various experimental manipulations. The study was approved by the Swedish Ethical Review Authority (https://etikprovningsmyndigheten.se) and conformed to the Declaration of Helsinki. After the completion of the experiment, one and a half hours in total, participants were compensated with one cinema ticket.

### Visual stimulation and HMD

**Fig 1:**
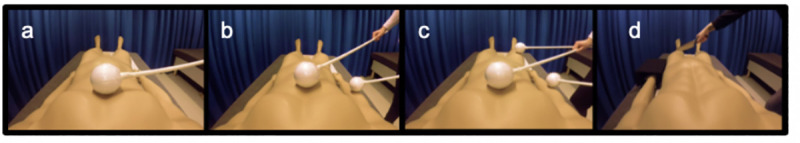
a-d. Visual stimulation. Display of an artificial mannequin’s body from the first-person perspective and the experimenter applying tactile stimulation to one (a), two (b), or three (c) body parts simultaneously. Visual stimulation was identical for both the synchronous and asynchronous conditions, the difference being the timing of the strokes applied to the participant’s real body. The participant lay on a bed with their head tilted forward and observed these videos through a head-mounted displays (HMD). Panel d displays the final scene comprising the knife in the SCR and illusion onset data collection (which was included for only the 1S, 3S, and 3A conditions; a still image of the mannequin’s body was instead presented at the end of the 1A, 2A and 2S conditions). Note. The presented images appear askew because they are monocular for illustrative purposes; a 3D binocular view (not askew) is achieved only within the HMD.

Visual stimulation comprised six pre-recorded videos of a trained experimenter using custom-built, plastic hand-held probes to apply tactile stimulation to the body of a life-sized male mannequin, which was presented from the natural (first-person) visual perspective in an anatomically natural posture (Fig 1A–1C). Visual stimulation was recorded using two GoPro cameras (GoPro HERO4 Silver, GoPro Inc., San Mateo, CA, USA) mounted above the mannequin’s body to provide two monocular recordings from the first-person perspective and edited using Final Cut Pro X Version 10.4.5. This software combined the two recordings to generate a three-dimensional stereoscopic image of the body when presented through the head-mounted display (HMD) set-up, for which we used an Oculus Rift DK 2 (California, USA). These steps helped to ensure that the false body spatially substituted for the subject’s own as much as possible when presented in the HMD, showing a three-dimensional humanlike body at a realistic angle, width and depth.

For the initial questionnaire data collection part of the experiment, the experimental videos containing visual stimulation were two minutes in duration. For the subsequent threat-evoked skin conductance response (SCR) and illusion onset time data collection, the experimental videos were elongated to two minutes and ten seconds to include the presentation of a knife or display a still image for an equivalent amount of time, depending on the experimental condition (see ‘Methods –Threat-evoked skin conductance response’ for details). Another distinction between the videos used for the questionnaire and the SCR/illusion onset time data collections was the visual inclusion of additional equipment in the latter to obtain threat-evoked SCRs (μS) (Biopac Systems Inc., MP150; Goleta, California, USA) and illusion onset times (seconds) (custom-made keypad). Two recording electrodes were attached to the middle and ring finger of the mannequin’s right hand, as they were for the participants, while its left hand was placed inside a black box (Fig 1D). Participants also placed their real left hand inside the box during the experiment, which contained a keypad with a single button to indicate illusion onset time (seconds). The box masked any visuomotor incongruency induced by real hand movements during button presses, which might otherwise have diminished the full-body ownership illusion [7,18,41].

### Visuotactile stimulation

Each experimental video, representing one of six experimental conditions (see ‘Methods –Experimental conditions’ for details), contained visual stimulation of sixteen tactile stimulations separated by a still image of the mannequin’s body, an inter-stimulus interval that ranged from four to nine seconds in duration (6.5 seconds on average). The frequency of visuotactile stimulation was some seconds slower than that in earlier studies [6,7,16] because 1) more prolonged periods of non-stimulation time were beneficial for the experimenter to accurately prepare, position, and align multiple stimuli for as close to perfect execution as possible (see further below).

Each tactile stimulus consisted of a white polystyrene ball with a diameter of eight centimetres attached to a stick of one metre for the experimenter to hold. The tactile stimuli shown in the experimental videos were the same as those used to stimulate the participant’s real body during the experiment. Each tactile stimulation covered a trajectory of fifteen centimetres on the corresponding body part(s) in the same direction and was always one second in duration. For both synchronous and asynchronous conditions, there was a total tactile stimulation time of sixteen seconds. The onset of the first visuotactile stimulation occurred at precisely twelve seconds; enough time for the single experimenter (S.O.) to initiate the video, position themself by the participant, and prepare to give the stimulation(s). To provide three simultaneous stimulations (Fig 1C), one probe was held in the experimenter’s left hand to stimulate the participant’s right arm. Meanwhile, for the experimenter’s right hand, the other two probes were attached together using elastic bands and positioned between the thumb and the index, plus the middle and the third finger, resembling the Musser-Stevens grip used by percussionists [44]. The two probes were held stable in this way, which enabled the experimenter to stimulate the participant’s trunk and right leg simultaneously. In terms of tactile stimulation force, the experimenter relied on the verbal report of pilot participants, who were asked to comment on whether the force was perceptually similar across the experimental conditions. They confirmed this to be the case, so the experimenter was satisfied that the force of the delivered stimuli was perceptually constant across the experimental conditions.

While participants observed the sequence of tactile stimulation being applied to the mannequin’s body, with their real body occluded from view by wearing the HMD, the experimenter applied either temporally synchronous (illusion) or asynchronous (control) tactile stimulation. For synchronous conditions, the timing and duration of each visuotactile stimulation was carefully controlled to match as closely as possible that witnessed by participants in the HMD. This was achieved using audio instructions created in Audacity Version 2.2.1, supplied only to the experimenter via noise-cancelling headphones. The audio instructions contained auditory cues pertaining to the onset and duration of the tactile stimulation, pure tones to announce stimulation(s) one second before onset, and white noise to indicate the duration of stimulation(s) in a vertical downwards trajectory. These cues were overlaid on a metronome with a tempo of 120 bpm such that two beats correspond with exactly 1 second in real time. The metronome was maintained audible in the track even during the period of white noise signalling the delivery of tactile stimulation, allowing for precise timing. For asynchronous conditions, the onset of this audio was delayed by two seconds, providing our stimulus onset asynchrony (SOA) of two seconds with respect to the synchronous condition. During asynchronous stimulation, the onset of visual stimulation always preceded tactile stimulation.

### Experimental conditions

The only factors that varied between the experimental conditions were 1) the synchronicity of the visuotactile stimulation and 2) the number of stimulations occurring simultaneously. Based on previous studies [5], we decided that stimulated body parts would comprise the trunk (one body-part condition), the trunk plus the right arm (two body-parts condition), or the trunk plus the right arm and the right leg (three body-parts condition) (Fig 1A–1C). Therefore, in a within-subjects 2 (stimulation synchronicity) × 3 (number of stimulations) design, the six experimental conditions were as follows: one body part with synchronous visuotactile stimulation (1S), one body part with asynchronous visuotactile stimulation (1A), two body parts with synchronous visuotactile stimulation (2S), two body parts with asynchronous visuotactile stimulation (2A), three body parts with synchronous visuotactile stimulation (3S) and three body parts with asynchronous visuotactile stimulation (3A).

### Questionnaire

The current study assessed participants’ subjective experiences using a 10-item questionnaire containing statements similar to those used previously in full-body ownership illusion studies [5,6] (Table 1). The questionnaire was distributed immediately after each experimental condition with statements arranged in a different order upon each presentation and always beginning with the header: “during the experiment, there were times when. . .”. Responses were made on a 7-point Likert scale ranging from ‘- 3’ to ‘+ 3’, describing the full range of agreeability from ‘strongly disagree’ to ‘strongly agree’, where ‘0’ represents uncertainty. The questionnaire contained items pertaining to participants’ experiences of referral of touch (Q1, Q2), five specific body-part ownership statements, individually referring to illusory ownership of the mannequin’s right arm (Q3), left arm (Q4), trunk (Q5), right leg (Q6) and left leg (Q7), and illusory full-body ownership (Q8), as well as control items to assess task compliance and suggestibility (Q9, Q10). Q8 was considered particularly important in this study since it represented the explicit experience of owning the entire artificial body. The referral of touch sensations was considered less important, since the statements did not discriminate which body parts sensed the touches; therefore, we did not emphasise Q1 or Q2 in our analyses.

**Table 1:** Questionnaire statements for the full-body ownership illusion including novel items for parts.

Item	Statement	Purpose
Q1	I felt the touch(es) given to the mannequin’s body	Referral of touch
Q2	It seemed as though the touch(es) I felt were caused by the probe(s) touching the mannequin’s body	Referral of touch
Q3	I felt as though the mannequin’s right arm were my arm	Body-part ownership
Q4	I felt as though the mannequin’s left arm were my arm	Body-part ownership
Q5	I felt as though the mannequin’s trunk were my trunk	Body-part ownership
Q6	I felt as though the mannequin’s right leg were my leg	Body-part ownership
Q7	I felt as though the mannequin’s left leg were my leg	Body-part ownership
Q8	I felt as though the mannequin’s whole body were my own body	Full-body ownership
Q9	I felt as though my real body were turning into a plastic body	Control
Q10	I felt naked	Control

### Threat-evoked skin conductance response

Threat-evoked skin conductance responses (SCRs) (μS) were also recorded in the current study, but only for three targeted conditions: 1S, 3S, and 3A. Given that only comparisons between these experimental conditions were necessary to assess our hypotheses regarding the SCR data (see Results –Threat-evoked skin conductance response for details) and since SCRs are known to diminish by habituation, with repeated exposure resulting in reduced responses [45], we chose to present the knife threat within only these three conditions rather than every condition. The knife threat comprised an identical five-second pre-recording of the experimenter presenting a large kitchen knife and moving it toward the thigh region of the mannequin’s left leg (Fig 1D), which was always preceded by the two-minute period of either synchronous or asynchronous visuotactile stimulation and was followed by five seconds of a still image of the mannequin’s body.

After conductive electrode gel (Biopac Systems Inc., Goleta, USA) was applied to the bottom surface of the third phalanges of the index and middle fingers, two recording electrodes were attached to the participant’s right hand (Biopac Systems Inc., Goleta, USA). Skin conductance (μS) was recorded continuously throughout the experiment using a Biopac MP150 (Biopac Systems Inc., Goleta, USA) and registered by the accompanying software (Acqknowledge 4.9). We collected the raw tonic signal at a sample rate of 100 Hz and analysed the data using the same manual extraction protocol described by [7] and for the same parameter of interest, the magnitude of threat-evoked SCRs. Using trigger codes flagging the onset of the knife threat, which were manually supplied to Acqknowledge during the running of the experiment upon a designated audio cue supplied only to the experimenter via noise-cancelling headphones, we analysed the SCR data offline. Each threat-evoked SCR was quantified as the magnitude in micro-siemens, μS, from the minimum to the maximum skin conductance level of the largest peak observed to onset a maximum of five seconds following the onset of the knife presentation [7,46]. Because these characteristic waveforms represent phasic changes in electrodermal activity that are time-locked to the onset of each knife stimulus, they are a well-founded representation of threat-evoked physiological responses [45,47] and, in turn, allow for a quantification of illusory ownership of the mannequin’s body [7,27,29].

First, an average response for each participant was calculated for each of the relevant experimental conditions: 1S, 3S, and 3A. As the data from two participants were excluded due to technical issues during signal acquisition and three more data sets were removed due to containing abnormally large values (an average SCR magnitude > 4.0 μS) [46], N = 43 for this analysis. We did not control for multiple comparisons in these specific analyses, since the number of planned comparisons was small (two) and we had a strong a priori hypothesis to expect the weakest SCR in the asynchronous condition (one-tailed: 3S – 3A) [7,16,17]. Post hoc, however, we extended this to the unplanned contrast, 1S – 3A.

### Full-body illusion onset time

In the same experimental blocks used to collect threat-evoked SCR data, we collected illusion onset time (seconds) for the full-body ownership illusion. There were a couple of responses provided during the asynchronous control conditions, but since these were both sparse and not of interest, we analysed only the responses whose onset times corresponded to the three synchronous conditions (1S, 2S, and 3S). This enabled us to examine whether the addition of stimulated body parts catalyses full-body ownership illusion onset (seconds).

Participants placed their left hand inside the box (Fig 1D) and their index finger over the response button within, in preparation to give a button press at their volition to indicate “the very first instance you experience the illusory sensation so as to feel as though the mannequin’s whole body were your own body”. Participants were reminded to press the button only once per experimental video and to refrain from pressing the button if they did not specifically perceive a full-body ownership illusion. Illusion onset times (seconds) were recorded relative to the first visuotactile stimulation, which occurred at precisely 12 seconds into the experimental video (see ‘Methods –Visuotactile stimulation’ for details).

### Procedures

Before the illusion was initiated, participants were instructed to lie on a bed with their head tilted approximately 30 degrees forward, supported by pillows, and to adopt a posture in which they could comfortably view their entire body. Participants spent a few minutes adjusting the HMD, showing only a still image of the mannequin’s body, for optimal clarity. They were then instructed to match their body posture to that of the mannequin as accurately and comfortably as possible, since maintaining a comfortable and similar bodily posture facilitates body ownership illusions via visuo-proprioceptive integration [48,49]. Participants were instructed to attend to the whole body rather than fixating on any particular body part. Participants also wore a pair of earplugs to eliminate sounds that could potentially influence the illusion experience, such as the sounds produced by the tactile stimuli contacting the real body [50]. After all the above preparations were completed and the participants confirmed that the instructions had been understood, the experimenter initiated the video containing visual stimulation. All instructions aforementioned were repeated to the participant before each experimental video (i.e., experimental block) began.

All 48 participants experienced the six experimental conditions (see ‘Methods –Experimental conditions’) three times: once for the initial questionnaire data collection, where the questionnaire was completed at the end of each experimental block, and twice for the threat-evoked SCR (μS)/illusion onset time (seconds) data collection. Questionnaire data collection always preceded the SCR/illusion onset time data collection; since the subjective questionnaire data were the main priority, we wanted the participants as naïve as possible when completing this. To circumvent the likely influence of order effects on each of our measures, we carefully counterbalanced the presentation of the six experimental conditions across participants and to both the questionnaire and the SCR/illusion onset time data collection. Using pseudo-randomisation of alternating blocks of synchronous and asynchronous conditions, a total of twelve possible orders of presentation for the experimental conditions were created. The total of twelve possible orders also provided the motivation to recruit a sample size of 48, affording four repetitions of each pseudo-randomisation across the participants.

As an exploratory side experiment, inspired by recent discussions about the relationship between body ownership and interoception [51–54], we also decided to take the opportunity to explore possible links between the magnitude of the full-body ownership illusion (Q8) and individual differences in ‘interoceptive sensibility,’ i.e., the self-evaluated assessment of one’s ability to sense internal bodily states [55,56]. To this end, participants completed the 18-item Body Awareness Questionnaire [57], which was given at the very end of the experiment. Since this aspect was non-central to the current study’s main focus, please find all information relating to the Body Awareness Questionnaire in ‘Supporting Information–Body Awareness Questionnaire’.

## Results

### Questionnaire data: Overview

**Fig 2:**
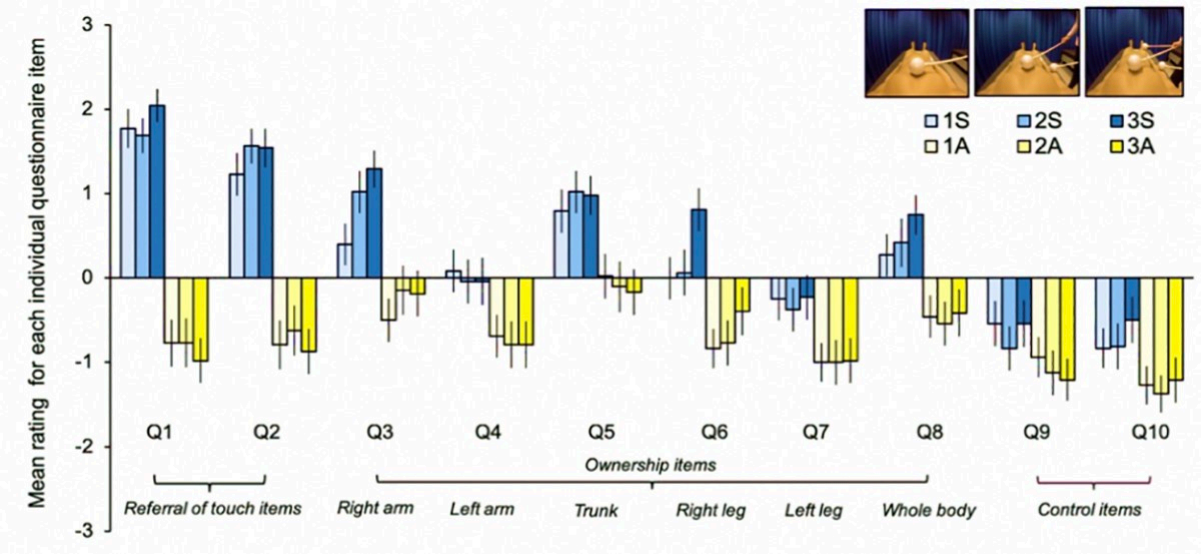
Mean ratings for each individual questionnaire item (Q1-Q10) across all six conditions. **N = 48.** Mean response to each questionnaire item, described by annotations within the figure, for conditions involving synchronous (blue) or asynchronous (yellow) visuotactile stimulation applied to one (lightest), two (intermediary), or three (darkest) body parts simultaneously. Error bars represent the standard error of the mean (SEM). Presented for illustrative purposes and for comparisons with earlier studies [7].

For descriptive purposes and to maintain consistency with earlier studies [7], mean ratings for all questionnaire items, which address referral of touch (Q1, Q2), illusory ownership of individual body parts (Q3 –Q7), illusory ownership of the entire artificial body (Q8), plus the control items (Q9, Q10), are presented below in Fig 2. Since all experimental questionnaire data pertaining to synchronous visuotactile stimulation are associated with positive ratings, all data pertaining to asynchronous visuotactile stimulation are associated with negative ratings, and control data are associated with negative ratings regardless of stimulation synchronicity. These results are consistent with the successful induction of an ownership illusion engaging the entire body on a descriptive level.

All inferential results for the planned comparisons are presented in Table 2, which contains uncorrected *p*-values and *p*-values (*p*_FDR_) corrected using the Benjamini-Hochberg false discovery rate [58], as well as a measure of effect size, *r* = *Z*/√N [59]. In ‘Supporting Information–Table S1’ in S1 File, there are additional data for the analyses comparing experimental against control items, as well as between the two control items themselves in ‘Supporting Information–Table S2’ in S1 File. All planned comparisons and post hoc tests involving questionnaire data were computed according to a 2-tailed hypothesis, and all *p*-values are reported in their original or, where multiple, corrected (*p*_FDR_) format.

**Table 2:** Subjective questionnaire results: Inferential statistics for all planned comparisons between synchronous and asynchronous stimulations and between the number of synchronous stimulations (one, two, or three) for all experimental questionnaire items (Q1-Q8).

Questionnaire item	*Z* score	*p* value	*p*_FDR_	*r*
**Q1 *χ*^2^(5) = 138.336, *p* < .001**				
**1S – 1A**	5.662	< .001	.002	.82
**2S – 2A**	5.333	< .001	.002	.77
**3S – 3A**	5.605	< .001	.002	.81
**2S – 1S**	2.123	.044	.0528	.31
**3S – 2S**	2.082	.044	.0528	.30
**3S – 1S**	0.503	.615	.615	.07
**Q2 *χ*^2^(5) = 110.543, *p* < .001**				
**1S – 1A**	4.991	< .001	.002	.72
**2S – 2A**	5.098	< .001	.002	.74
**3S – 3A**	4.898	< .001	.002	.71
**2S – 1S**	1.758	.119	.1785	.25
**3S – 2S**	0.61	.951	.1968	.09
**3S – 1S**	1.489	.164	.951	.21
**Q3 *χ*^2^(5) = 67.840, *p* < .001**				
**1S – 1A**	3.856	< .001	.0015	.56
**2S – 2A**	4.319	< .001	.0015	.62
**3S – 3A**	4.544	< .001	.0015	.66
**2S – 1S**	2.850	.005	.006	.41
**3S – 2S**	1.077	.281	.281	.16
**3S – 1S**	3.500	< .001	.0015	.51
**Q4 *χ*^2^(5) = 27.422, *p* < .001**				
**1S – 1A**	3.119	.004	.008	.45
**2S – 2A**	3.260	.004	.008	.47
**3S – 3A**	3.130	.004	.008	.45
**2S – 1S**	0.388	.979	.979	.06
**3S – 2S**	0.026	.979	.979	.004
**3S – 1S**	0.168	.979	.979	.02
**Questionnaire item**	***Z* score**	***p* value**	***p***_**FDR**_	***r***
**Q5 *χ*^2^(5) = 52.665, *p* < .001**				
**1S – 1A**	3.043	.004	.003	.44
**2S – 2A**	4.138	< .001	.003	.60
**3S – 3A**	3.773	< .001	.008	.54
**2S – 1S**	1.475	.21	.315	.21
**3S – 2S**	0.182	.875	.875	.03
**3S – 1S**	1.044	.355	.426	.15
**Q6 *χ*^2^(5) = 63.083, *p* < .001**				
**1S – 1A**	3.686	< .001	.002	.53
**2S – 2A**	3.610	< .001	.002	.52
**3S – 3A**	4.503	< .001	.002	.55
**2S – 1S**	0.484	.628	.628	.07
**3S – 2S**	2.930	.004	.0048	.42
**3S – 1S**	3.283	.002	.003	.47
**Q7 *χ*^2^(5) = 36.895, *p* < .001**				
**1S – 1A**	3.752	< .001	.003	.54
**2S – 2A**	3.344	.002	.004	.48
**3S – 3A**	3.608	< .001	.003	.52
**2S – 1S**	0.404	.743	.743	.06
**3S – 2S**	0.882	.567	.743	.13
**3S – 1S**	0.328	.743	.743	.05
Q8* *χ*^2^(5) = 59.527, *p* < .001				
**1S – 1A**	3.216	.002	.004	.46
**2S – 2A**	3.376	.002	.004	.49
**3S – 3A**	4.212	< .001	.004	.61
**2S – 1S**	0.687	.492	.492	.10
**3S – 2S**	1.393	.163	.1956	.20
**3S – 1S**	2.032	.043	.0645	.29

Post hoc test results are reported within the main text.

### Full-body ownership

**Fig 3:**
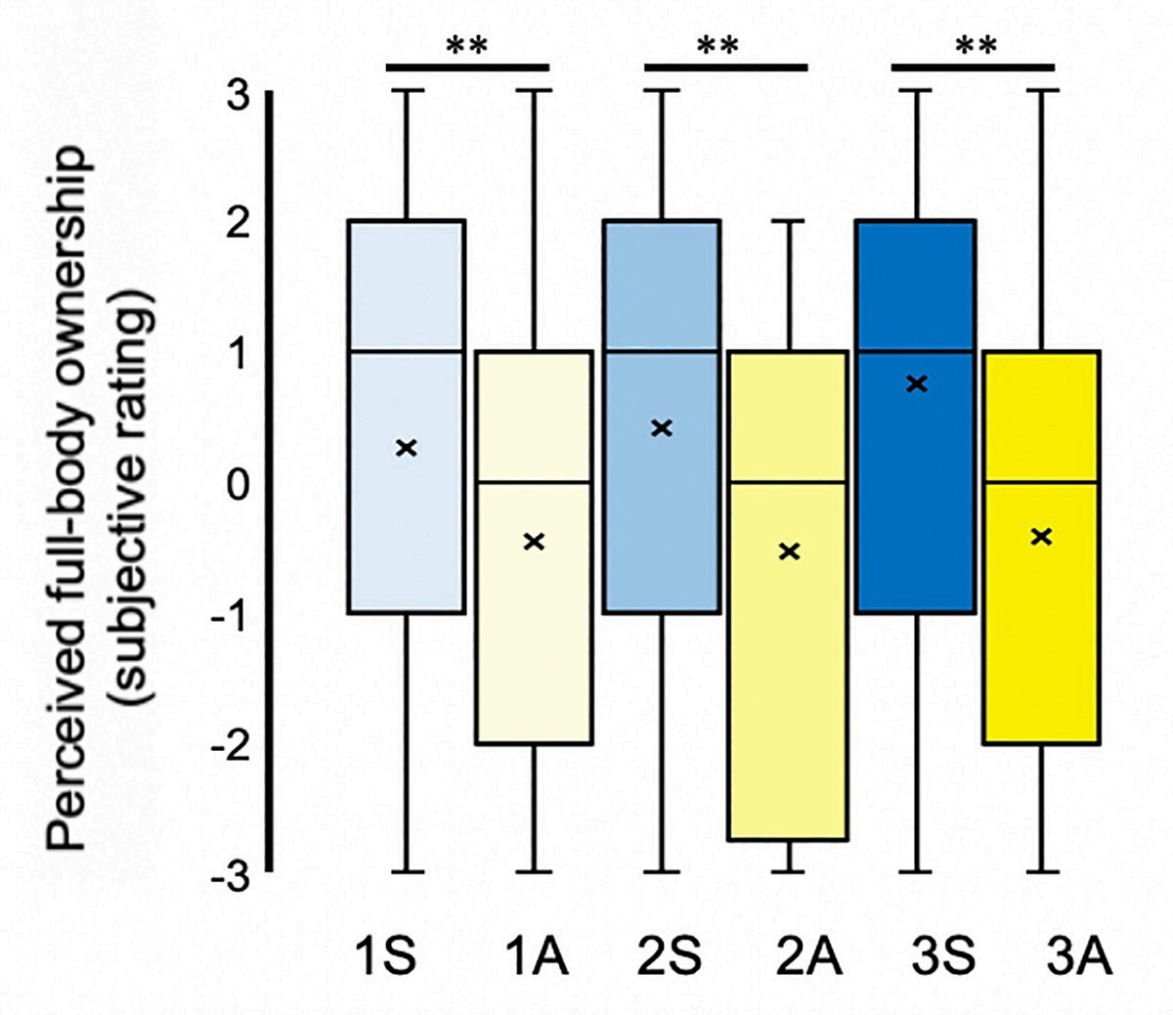
Illusory ownership for the mannequin’s whole body. **N = 48.** Perceived ownership for the entire artificial body across both synchronous (blue) and asynchronous (yellow) visuotactile stimulation to one (lightest), two (intermediary), and three (darkest) body parts simultaneously. The mean and median values are represented by the • and the straight line within the boxplot, respectively. Note. *** indicates significance at *p* < .001, ** indicates significance at *p* < .01 and * indicates significance at *p* < .05 after Benjamini-Hochberg FDR correction.

The results for Q8, the critical questionnaire item referring to illusory full-body ownership, are displayed in Fig 3 and presented in Table 2; Q8.

We analysed the effects of the 2 x 3 design on illusory full-body ownership ratings (Q8) using linear mixed models in R Version 4.0.2 [60]. Specifically, illusory full-body ownership ratings (Q8) were analysed in a linear mixed model using both the lmer function of the lme4 package [61] and the rlmer function of the robustlmm package [62], where the latter is more robust to violations of the assumptions of the normal distribution. In both cases, the model (M1) specified stimulation synchronicity (synchronous versus asynchronous), the number of stimulations (one, two versus three), and the 2 x 3 interaction as fixed effects, while the individual participant (N = 48) depicted a random effect. The 2 x 3 interaction term is particularly important for the focus of the current study, since it examines whether the combination of synchronous stimulation and its delivery to multiple body parts significantly facilitates the illusory percept of full-body ownership, which is more meaningful than a main effect of the number of stimulated body parts, per se. We additionally calculated an interaction term manually by comparing the difference in full-body ownership ratings between synchronous and asynchronous stimulation for the most extreme conditions, the stimulation of three body parts versus one, using the contrast [(3S – 3A)–(1S – 1A)] in a Wilcoxon signed-rank test. We also tested this contrast using a Bayesian paired t-test (default prior) conducted in JASP, Version 0.9.2.

For M1, the linear mixed model estimate representing stimulation synchronicity was significant (B = 0.733, SE: 0.201, *t* = 3.649, *p* < .001), confirming that the experimental manipulation of stimulation synchronicity was an important predictor of illusory full-body ownership ratings. However, the model estimates representing both the number of stimulations and the 2 x 3 interaction term were found to be non-significant (B = 0.021, SE: 0.120, *t* = 0.189, *p* = .850 and B = 0.219, SE: 0.156, *t* = 1.407, *p* = .160, respectively). Very similar results were obtained when implementing the robust linear mixed model (stimulation synchronicity: B = 0.767, SE: 0.185, *t* = 4.142, *p* < .00; number of stimulations: B = 0.016, SE: 0.101, *t* = 0.159, *p* = .874; 2 x 3 interaction: B = 0.196, SE: 0.143, *t* = 1.370 *p* = .177). Therefore, model evidence suggests that illusory full-body ownership ratings depended on the synchronicity of the visuotactile stimulation and not the number of stimulated body parts. Consistent with this, our planned comparisons revealed that synchronous visuotactile stimulation resulted in significantly increased illusory full-body ownership ratings compared to asynchronous visuotactile stimulation, while there were no differences between conditions varying by the number of synchronously stimulated body parts (Table 2; Q8). Similar to the linear mixed model estimate, the result of the Wilcoxon signed-rank test examining the interaction via the contrast [(3S – 3A)–(1S – 1A)] was non-significant (*Z* = 1.651, *p* = .099), and the Bayes factors associated with this contrast suggested greater support for the null (BF_01_ = 1.628) rather than the alternate hypothesis (BF_10_ = 0.614). However, evidence in favour of either hypothesis was ‘anecdotal’ [63].

Next, we decided to specify four additional linear mixed models: M0 to represent the null hypothesis (without any fixed effects); M2, defining only stimulation synchronicity as a fixed effect; M3, defining both stimulation synchronicity and the number of stimulations as fixed effects; and M4, defining only the number of stimulations as a fixed effect. For all models (M0 –M4), the individual participant (N = 48) represented the random effect. Using ANOVA, we compared model fit between the five different models using (1) a stepwise intake of predictors (fixed effects) starting with stimulation synchronicity and (2) a stepwise intake of predictors starting with the number of stimulations. Compared to the null model (M0), the model specifying only stimulation synchronicity as a fixed effect (M2) provided a significantly improved fit with our data (*X*^2^(1) = 50.112, *p* < .001). Neither including the number of stimulations (M3) nor both the number of stimulations and the 2 x 3 interaction (M1) as fixed effects led to any significant changes in model fit from M2 (*X*^2^(1) = 2.80, *p* = .094; *X*^2^(1) = 1.995, *p* = .158). Meanwhile, specifying only the number of stimulations as a fixed effect (M4) did not lead to a significant change in model fit compared to the null model, M0 (*X*^2^(1) = 2.270, *p =* .132). The estimate for the 2 x 3 interaction term was non-significant in M1 (see above). and its inclusion in the linear mixed model did not statistically improve model fit from M2, which specified only stimulation synchronicity as a fixed effect. The findings fail to support any significant enhancements in illusory full-body ownership ratings by stimulating multiple body parts simultaneously, a notion reinforced by our planned pairwise comparisons for illusory full-body ownership ratings, which showed that 2S - 1S, 3S - 2S, and 3S -1S were all non-significant (*p*_FDR_>0.05; Table 2; Q8).

### Body-part ownership

**Fig 4:**
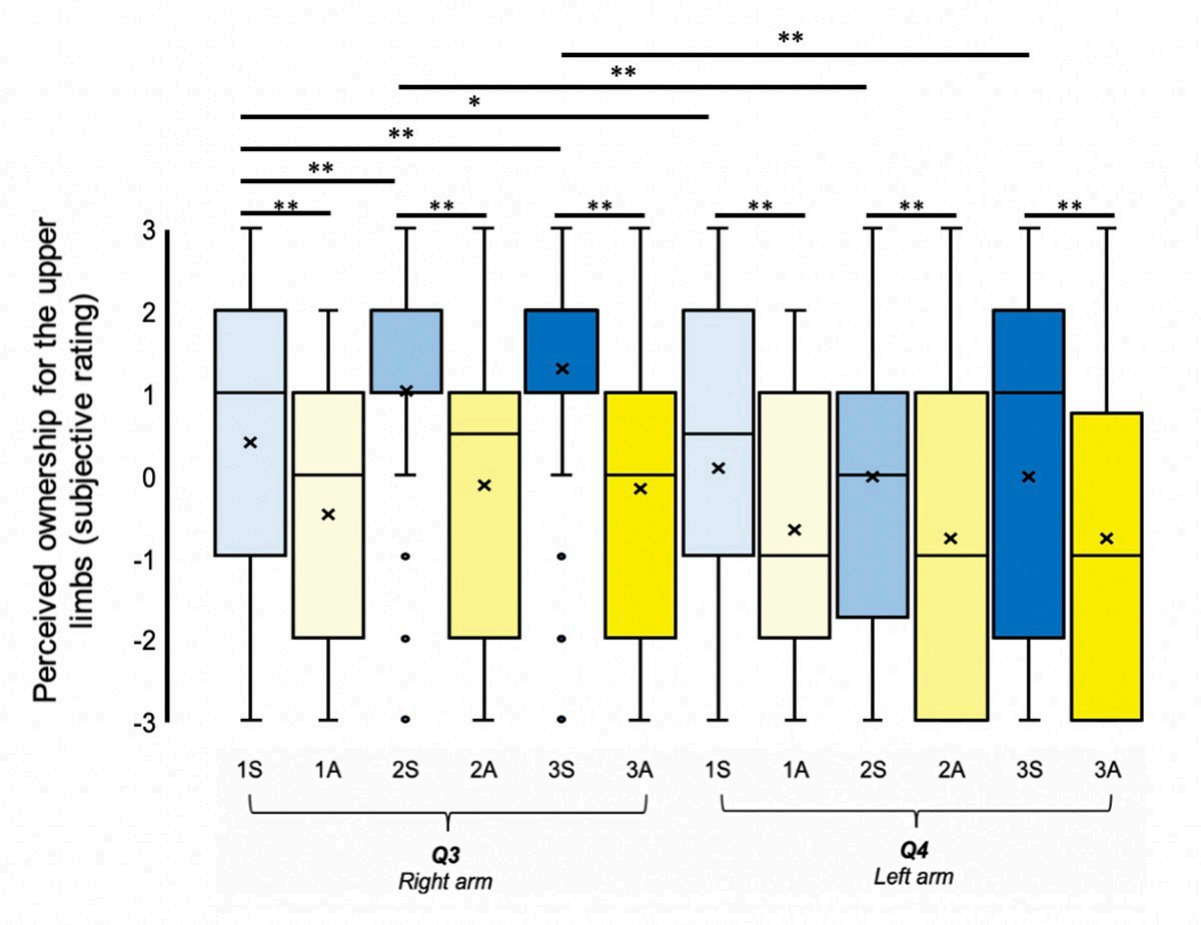
Illusory ownership for the mannequin’s upper limbs. **N = 48.** Perceived ownership for the right arm (Q3) and left arm (Q4) across both synchronous (blue) and asynchronous (yellow) visuotactile stimulation to one (lightest), two (intermediary), and three (darkest) body parts simultaneously. The mean and median values are represented by the x and the straight line within the boxplot, respectively. Note. *** indicates significance at *p* < .001, ** indicates significance at *p* < .01 and * indicates significance at *p* < .05 after Benjamini-Hochberg FDR correction.

**Fig 5:**
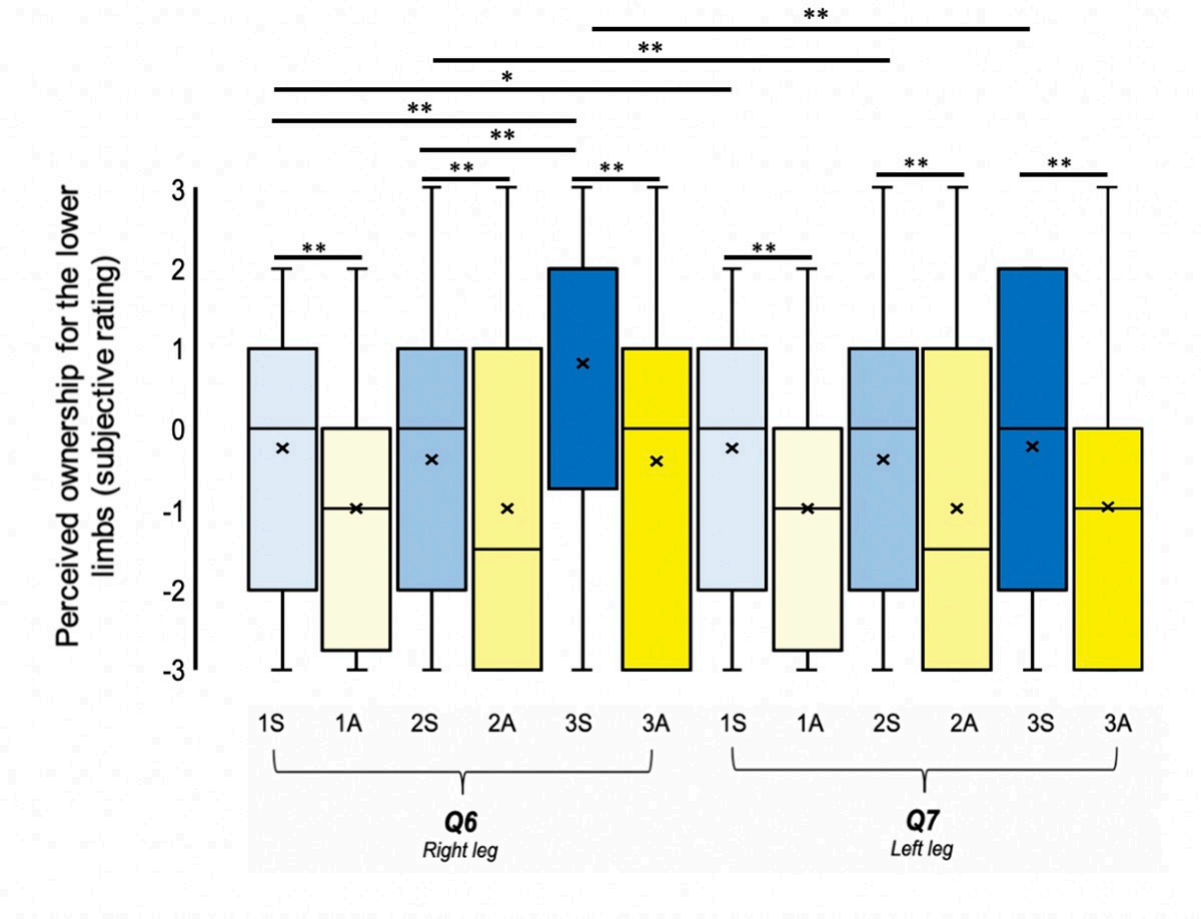
Illusory ownership for the mannequin’s lower limbs. **N = 48.** Perceived ownership for right leg Q6 and left leg Q7 across both synchronous (blue) and asynchronous (yellow) visuotactile stimulation to one (lightest), two (intermediary), and three (darkest) body parts simultaneously. The mean and median values are represented by the x and the straight line within the boxplot, respectively. Note. *** indicates significance at p < .001, ** indicates significance at p < .01 and * indicates significance at p < .05 after Benjamini-Hochberg FDR correction.

**Fig 6:**
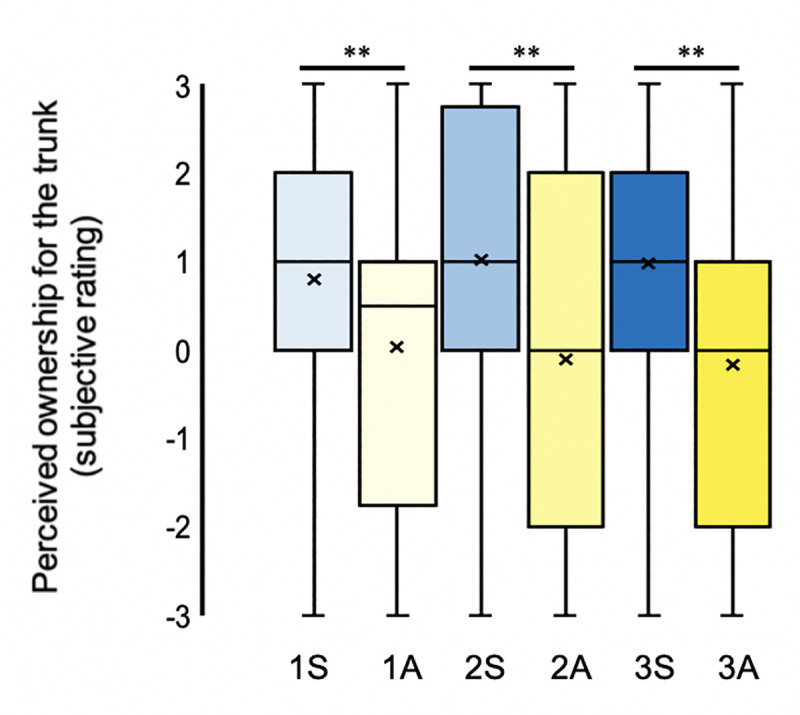
Illusory ownership for the mannequin’s trunk. **N = 48.** Perceived ownership of the trunk, Q5, across both synchronous (blue) and asynchronous (yellow) visuotactile stimulation to one (lightest), two (intermediary), and three (darkest) body parts simultaneously. The mean and median values are represented by the x and the straight line within the boxplot, respectively. Note. *** indicates significance at *p* < .001, ** indicates significance at *p* < .01 and * indicates significance at *p* < .05 after Benjamini-Hochberg FDR correction.

Synchronous visuotactile stimulation of the mannequin’s body resulted in significantly increased illusory ownership ratings for all of the mannequin’s individual body parts compared to asynchronous visuotactile stimulation (Figs 4–6; Table 2; Q3 –Q7). This finding included body parts that had received visuotactile stimulation at some point during the study (the trunk in condition 1S; the trunk, plus the right hand in condition 2S; the trunk, the right arm, plus the right leg in condition 3S) as well as the left limbs, which were never stimulated. Moreover, the results of a regression analysis (concatenating 1S, 2S, and 3S) revealed that the magnitude of illusory ownership ratings (synchronous minus asynchronous) for non-stimulated body parts could be predicted by the magnitude of ratings for stimulated body parts, suggesting that there was a bottom-up “spread of ownership” [5,7] between stimulated and non-stimulated body parts during the full-body ownership illusion (*χ*^2^(26, N = 48) = 93.838, *p* < .001, pseudo-*R*^2^ (McFadden) = .129). However, only 12.9% of the variance in illusory ownership ratings for non-stimulated body parts could be explained by the variance in illusory ownership ratings for stimulated body parts. Since the shared variance is small (12.9%), factors other than illusory ownership for stimulated body parts must contribute to the perception of illusory ownership for non-stimulated body parts.

Similar to the results for illusory full-body ownership, the magnitude of the “spreading effect” between stimulated and non-stimulated body parts was unchanged by increasing the number of stimulated body parts. For example, shared variance for regression analyses ran separately for one, two and three body-part conditions between stimulated and non-stimulated (left) body parts (synchronous–asynchronous ratings) and was not found to increase with the stimulation of additional body parts (1 body part: *χ*^2^(7, N = 48) = 24.722, *p* = .001, pseudo-*R*^2^ (McFadden) = .122; 2 body parts: *χ*^2^(11, N = 48) = 20.517, *p* = .039, pseudo-*R*^2^ (McFadden) = .112; 3 body parts: *χ*^2^(16, N = 48) = 38.697, *p* = .001, pseudo-*R*^2^ (McFadden) = .192). Moreover, illusory ownership ratings for the left body parts, which were never stimulated in this study, were not significantly different across 1S, 2S and 3S (Table 2; Q4 and Q7), and there were no significant differences between the conditions using synchronous minus asynchronous ratings for these body parts (2–1 body parts: *Z* = 0.290, *p* = .772, *r* = .04; 3–2 body parts: *Z* = 0.788, *p* = .431, *r* = .11; 3–1 body parts: *Z* = 0.533, *p* = .594, *r* = .08). The only significant increases in body part ownership between conditions 1S, 2S, and 3S were associated with the specific body part(s) receiving synchronous visuotactile stimulation (see further below).

We found that illusory ownership ratings for individual body parts were significantly increased when the body parts in question were stimulated synchronously, not only compared to when they were stimulated asynchronously but also compared to when they were not stimulated (Figs 4–6). Therefore, for both 2S and 3S, conditions in which the right arm received synchronous stimulation, illusory ownership ratings for the mannequin’s right arm were significantly higher than for 1S, the condition in which the right arm received no stimulation (Fig 4, Table 2; Q3). Similarly, for 3S, the condition in which the right leg received synchronous stimulation, illusory ownership ratings for the mannequin’s right leg were significantly higher than for either 2S or 1S, conditions in which the right leg received no stimulation (Fig 5, Table 2; Q6). These findings support the hypothesis that synchronous visuotactile stimulation boosts body-part ownership for body parts receiving synchronous visuotactile stimulation. However, once synchronously stimulated, there is a ceiling effect in illusory ownership ratings for body parts, i.e., the synchronous stimulation of both the right arm and the right leg in condition 3S does not increase illusory body part ownership ratings for the mannequin’s right arm beyond that observed in 2S. Additionally, consistent with this hypothesis, no significant changes were observed in illusory ownership for the mannequin’s trunk, the only body part to receive synchronous visuotactile stimulation during all three synchronous conditions (Fig 6, Table 2; Q5). Unlike illusory full-body ownership ratings and illusory ownership ratings for non-stimulated body parts, illusory ownership ratings for stimulated body parts were affected by whether one, two or three body parts were synchronously stimulated.

#### Left versus right body-part ownership

While we observed similar levels of illusory body-part ownership for the mannequin’s left limbs across the three synchronous conditions (Table 2; Q4, Q7), we found marked asymmetry in illusory ownership ratings between the left versus the right half of the mannequin’s body (‘hemibody’) (e.g., Fig 2). Using Friedman’s tests and the Wilcoxon signed-rank test, we assessed the differences in illusory body-part ownership ratings between the right and the corresponding left body-part for each of the three synchronous conditions.

Regarding the upper limbs (Fig 4), there were significant differences between participants’ illusory ownership ratings for the mannequin’s left versus right arm across the three synchronous conditions (χ^2^(2) = 57.332, p < .001). The Wilcoxon signed-rank test revealed that illusory ownership ratings for the mannequin’s right arm were significantly higher than illusory ownership ratings for the mannequin’s left arm, both when neither limb received any stimulation (1S: *Z* = 2.354, *p* = .019, *p*_FDR_ = .019, *r* = .34) and, more expectedly, when the right arm received synchronous visuotactile stimulation (2S: *Z* = 4.533, *p* < .001, *p*_FDR_ = .0015, *r* = .65 and 3S: *Z* = 4.692, *p* < .001, *p*_FDR_ = .0015, *r* = .68). Likewise, for the lower limbs (Fig 5), participants rated illusory ownership to significantly different degrees across the three synchronous conditions (*χ*^2^(2) = 32.846, *p* < .001). The Wilcoxon signed-rank test also revealed significantly increased illusory ownership ratings for the mannequin’s right leg compared to the mannequin’s left leg when neither leg received stimulation (1S: *Z* = 1.983, *p* = .047, *p*_FDR_ = .047, *r* = .29 and 2S: *Z* = 2.946, *p* = .003, *p*_FDR_ = .0045, *r* = .43) and, more expectedly, when the right leg received synchronous visuotactile stimulation (3S: *Z* = 4.692, *p* < .001, *p*_FDR_ = .003, *r* = .68). Therefore, a left-right asymmetry in illusory body-part ownership ratings, which favoured the mannequin’s right hemibody compared to the mannequin’s left hemibody, persisted across all loads of multisensory stimulation (1S, 2S and 3S) and for both the upper (Fig 4) and the lower (Fig 5) limbs of the mannequin. Since we observed a general trend for participants to ascribe higher illusory ownership ratings for the mannequin’s right compared to the left hemibody (i.e., also during some of the asynchronous conditions; see ‘Supporting Information –Left vs. right illusory ownership for asynchronous conditions’), we reconducted these analyses using synchronous minus asynchronous ratings. The left-right difference remained significant for the 2- and 3-body-part conditions (*Z* = 3.456, *p* = .001; *Z* = 2.590, *p* = .01) but was no longer significant for the 1-body-part condition (*Z* = 0.783, *p* = .433), suggesting that the asymmetrical effect was mainly driven by the synchronous stimulation of the right-sided body-part(s).

### Part-to-whole ownership relationships

**Fig 7:**
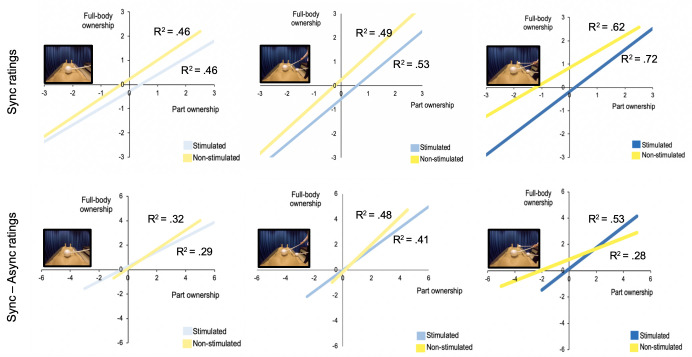
a-f. Rated illusory ownership for the whole body versus stimulated and non-stimulated parts in 1S, 2S, 3S (a-c) and for the difference between synchronous and asynchronous stimulation for 1, 2, and 3 body parts. N = 48. Positive linear relationships between subjective ownership for the entire artificial body and its parts, both for synchronously stimulated body part(s) (blue) (1S: trunk; 2S: trunk and right arm; 3S: trunk, right arm and right leg) and non-stimulated (neither synchronous nor asynchronous) body parts (yellow) (1S: right arm, left arm, right leg and left leg; 2S: left arm, left leg and right leg; 3S: left arm and left leg. Conditions 1S, 2S, and 3S are represented by panels a, b and c, respectively, while the difference in ratings between 1S – 1A, 2S – 2A, and 3S – 3A are represented by panels d, e and f, respectively. In the cases of multiple body parts, an average rating was formed for their comparison with illusory full-body ownership (singular item, Q8). All correlations were found to be significant at p < .001. After concatenating condition type (1S, 2S, 3S) and subtracting the corresponding asynchronous ratings (sync-async), regression analyses for all body parts, stimulated body parts and non-stimulated body parts were also significant (at least p < .01).

Using Spearman’s rank correlations, we analysed the relationships between body-part ownership (for both the stimulated and non-stimulated body parts) and full-body ownership. This gave us an indication of whether they describe related perceptual phenomena (irrespective of causality) in each of the three synchronous conditions, as well as whether the correlation co-efficient (i.e., the strength of this relationship) changes with respect to the number of stimulated body parts (one, two or three). Consistently, strong significant positive correlations were identified between illusory ownership ratings for stimulated body part(s) (calculated as average ratings of the relevant questions for each condition; 1S = Q5; S2 = (Q3+Q5)/2; 3S = (Q3+Q5+Q6)/3) and full-body ownership ratings (Q8): 1S: *r*_s_ = .68, *p* < .001; 2S: *r*_s_ = .73, *p* < .001 and 3S: *r*_s_ = .85, *p* < .001. Similarly, strong positive correlations were identified between illusory ownership ratings for non-stimulated body part(s) (1S = (Q3+Q4+Q6+Q7)/4; 2S = (Q4+Q6+Q7)/3; 3S = (Q4+Q7)/2) and the whole body (Q8): 1S: *r*s = .68, *p* < .001; 2S: *r*_s_ = .70, *p* < .001 and 3S: *r*_s_ = .79, *p* < .001 (Fig 7A–7C, respectively). Moreover, these correlations retained significance when the difference between synchronous and asynchronous ratings was used instead: 1S: stimulated *r*s = .54, *p* < .001, non-stimulated *r*s = .57, *p* < .001; 2S: stimulated *r*s = .64, *p* < .001, non-stimulated *r*s = .69, *p* < .001; S3: stimulated *r*s = .73, *p* < .001, non-stimulated *r*s = .53, *p* < .001. Therefore, the greater the subjective magnitude of illusory ownership for both stimulated and non-stimulated body part(s), the greater the subjective magnitude of illusory ownership for the whole body.

In light of these results, we also computed post hoc ordinal regression analyses on the synchronous minus asynchronous body-part ownership ratings, concatenating the conditions 1S, 2S and 3S. This gave us an indication of whether illusory ownership of body parts (all, stimulated and non-stimulated) could predict the magnitude of the resulting full-body ownership illusion. These regression analyses were significant in all cases: averaged ratings for all parts-to-whole, *χ*^2^(59, N = 48) = 300.054, *p* < .001, pseudo-*R*^2^ (McFadden) = .575 (shared variance, 57.5%); averaged ratings for stimulated parts-to-whole, *χ*^2^(26, N = 48) = 153.964, *p* < .001, pseudo-*R*^2^ (McFadden) = .295 (shared variance, 29.5%); averaged ratings for non-stimulated parts-to-whole, *χ*^2^(24, N = 48) = 80.058, *p* < .001, pseudo-*R*^2^ (McFadden) = .153 (shared variance, 15.3%). Therefore, overall, approximately half of the variance (57.5%) in illusory full-body ownership ratings could be attributed to the variance in illusory ownership for all body parts (both stimulated and non-stimulated). Moreover, by specifying illusory full-body ownership ratings as the regressor and illusory ownership ratings for non-stimulated body parts as the dependent variable, we also examined whether full-body ownership may facilitate the “spread of ownership”, instead of the opposite relationship tested above (the “spread of ownership” facilitating full-body ownership). Although the analysis revealed a significant relationship, *χ*^2^(9, N = 48) = 67.002, *p* < .001, pseudo-*R*^2^ (McFadden) = .09, with a lower shared variance of 9%, it could be the case that illusory full-body ownership drives the “spread of ownership” to a lesser extent than the “spread of ownership” drives illusory full-body ownership. However, since this interpretation is based upon the relative shared variance, it should be interpreted with caution.

### Threat-evoked skin conductance response

The threat-evoked SCR data (μS) were analysed using the Wilcoxon signed-rank test, as our two planned comparisons were designed to compare only the magnitude of threat-evoked SCRs (μS) between synchronous and asynchronous visuotactile stimulation (3S – 3A; one-tailed) to assess the basic effect of the illusion and to examine the effect of increasing the number of synchronously stimulated body parts from one to three (1S – 3S; two-tailed).

**Fig 8:**
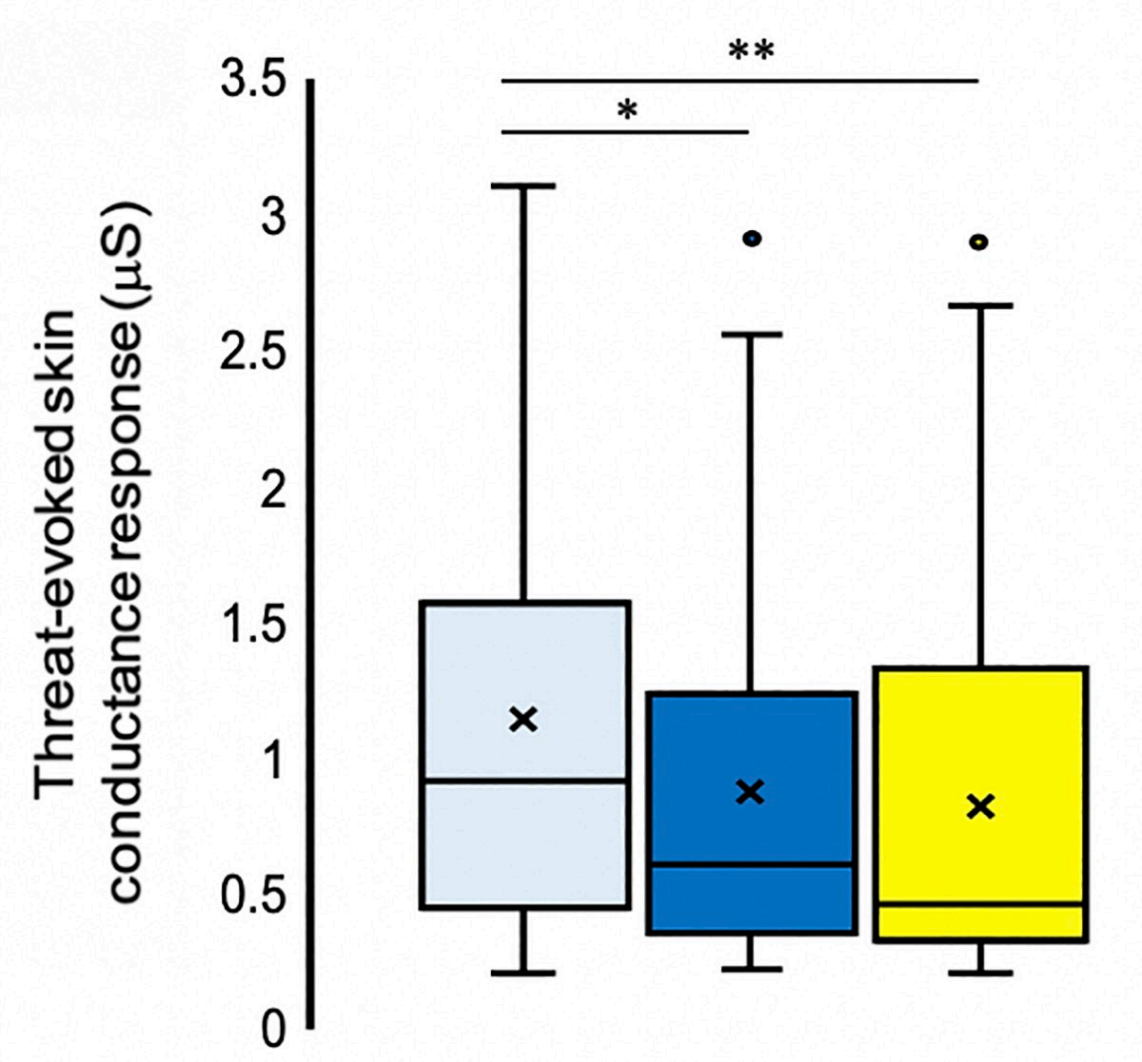
Threat-evoked skin conductance responses (μS). **N = 43.** Threat-evoked SCRs following conditions of synchronous visuotactile stimulation applied to three (dark blue) and one body part (light blue) and asynchronous (control) stimulation to three body parts (yellow). The mean and median values are represented by the x and the straight line of the boxplot, respectively. Note. ** indicates significance at *p* < .01 and * indicates significance at *p* < .05.

**Fig 9:**
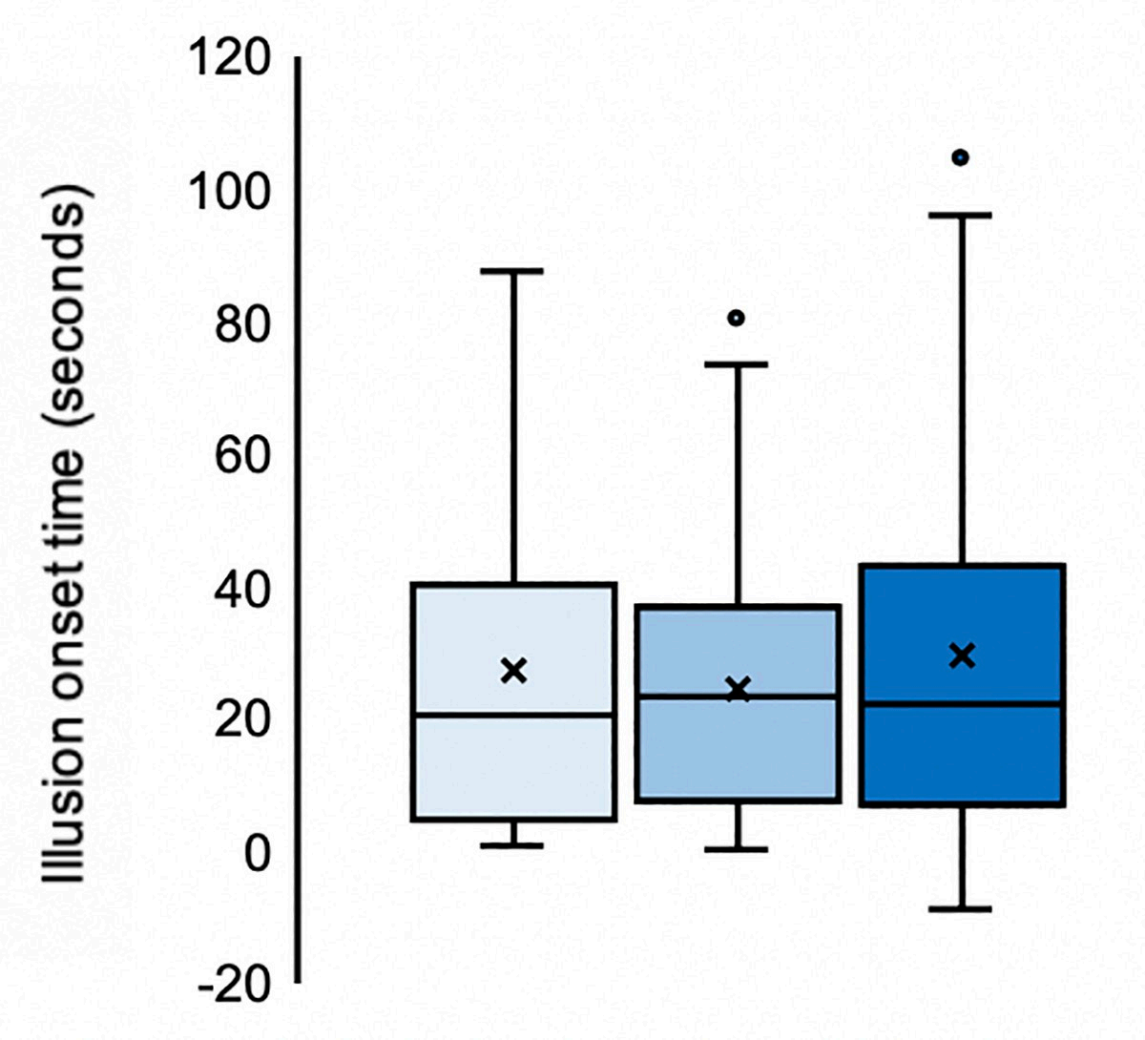
Full-body illusion onset time (seconds). **N = 33.** Illusion onset times (seconds) averaged over the two repetitions for each two-minute stimulation of conditions involving synchronous visuotactile stimulation to one, two, or three body parts simultaneously (lightest–darkest). The mean and median values are represented by the x and the straight line within the boxplot, respectively. For clarity and rounded to the nearest second: 1S: mean = 28 seconds, median = 21 seconds; 2S: mean = 25 seconds, median = 24 seconds and 3S: mean = 30 seconds, median = 22 seconds. T_0_ = the onset of the very first visuotactile stimulation, which was preceded by 12 seconds of visuo-proprioceptive stimulation as the experimenter prepared to apply the stimulations to the participants’ real body.

A statistically significant difference was found between threat-evoked SCRs (μS) for 3S – 1S (two-tailed: *Z* = -2.137, *p* = .033, *r* = .33), but unexpectedly, the direction of responses was significantly greater for condition 1S than for condition 3S (Fig 8). Against our a priori expectations and the questionnaire results described above, we also did not observe significantly stronger threat-evoked SCRs (μS) for the condition with synchronous visuotactile stimulation than for the condition with asynchronous visuotactile stimulation (one-tailed: 3S – 3A: *Z* = 0.537, *p* = .296, *r* = .08). Therefore, as a ‘sanity check’, we decided to analyse the contrast 1S – 3A post hoc, and one-tailed, due to our strong a priori hypothesis regarding the direction of any difference and because the 1S condition specifically has been used before [7]. This analysis did reveal a significant difference and in the expected direction (*Z* = 2.985, *p* = .002, *r* = .53). Therefore, there must have been something peculiar about the experimental condition 3S in particular that weakened threat-evoked SCRs. We speculate that this peculiarity may be the product of using the mannequin’s left leg as the threat-targeted body part combined with the stimulation of right-sided limbs in condition 3S (see Discussion for details). In summary, the current SCR findings (Fig 9) produced mixed evidence in support of a successful full-body ownership illusion.

### Full-body illusion onset time

By analysing illusion onset time (seconds) between 1S, 2S, and 3S, we investigated whether the addition of synchronously stimulated body parts decreased the rate at which an explicit illusory whole-body percept emerged. Due to the response rate of 69%, N = 33 for this analysis. The response rate of 69% (33/48) is roughly akin to that reported for the rubber hand illusion in [64] for approximately 70–75% of recruited samples. We first analysed the data from these 33 participants, who pressed the button at least once across each experimental condition's two repeats. Second, we used only the data of consistent responders, who supplied all responses in both repetitions of the experimental conditions (N = 20).

Friedman’s tests returned no evidence of significant differences in illusion onset time (seconds) between any of the three synchronous conditions (N = 33: *χ*^2^(2) = 0.424, *p* = .809; N = 20: *χ*^2^(2) = 1.3, *p* = .522). Therefore, there was no evidence of any effect of increasing the number of stimulated body parts on full-body ownership illusion onset time (seconds) in the current study. These findings (N = 33) are summarised in Fig 9.

We further decided to correlate the subjective magnitude of the full-body ownership illusion (Q8) with the reported onset time (seconds) to examine whether there was any significant relationship between the subjective strength of the full-body ownership illusion and its temporal onset. Spearman’s rank correlations were non-significant for all three conditions (N = 33: 1S: *r*_s_ = -.182, *p*_FDR_ = .467; 2S: *r*_s_ = -.106, *p*_FDR_ = .557 and 3S: *r*_s_ = -.238, *p*_FDR_ = .467 and N = 20: 1S: *r*_s_ = -.280, *p*_FDR_ = .232; 2S: *r*_s_ = -.084, *p*_FDR_ = .725 and 3S: *r*_s_ = -.516, *p*_FDR_ = .06). Therefore, the results do not support any significant relationship between the subjective magnitude of the full-body ownership illusion and its rate of onset in the current study.

## Discussion

The present experiment set out primarily to investigate whether simply increasing the number of synchronously stimulated body parts significantly increases perceived full-body ownership during a full-body ownership illusion [7]. Should increasing the number of synchronously stimulated body parts contribute significant enhancements to illusory full-body ownership, it could be suggested that full-body ownership reflects nothing more than the summation of ownership across constituent body parts. However, the results of both the linear mixed modelling and planned comparisons revealed that illusory full-body ownership ratings (Q8) were not significantly enhanced by converging synchronous multisensory stimulation across multiple body parts simultaneously. Significant effects in illusory full-body ownership ratings concerned only the synchronicity of the visuotactile stimulation. Moreover, although we observed significant relationships between illusory body-part and full-body ownership ratings, only approximately half (57.5%) of the variance in illusory full-body ownership ratings could be explained by variance in illusory ownership ratings for all of the body-parts. This suggests that another explanatory variable must be involved, which can account for the remaining variance in illusory full-body ownership ratings. This variable likely reflects cognitive processes specific to the full-body ownership percept and involves neural substrates unique to those that represent the owned body parts [5,6]. The notion that separate neural substrates represent information about body parts and whole bodies is also supported by neuroimaging studies that examine their visual representations [65,66]. An apt explanation comes from the influential Gestalt psychologist Kurt Koffka [67], who famously stated, “It has been said: the whole is more than the sum of its parts. It is more correct to say that the whole is something else than the sum of its parts, because summing up is a meaningless procedure, whereas the whole-part relationship is meaningful”. In line with this conjecture in the visual sciences, in own-body perception, the summation of illusory body-part ownership does not appear to provide a complete account of illusory full-body ownership. However, this does not discount meaningful relationships between body part and full-body ownership perception.

Former studies investigating the full-body ownership illusion [5–7] have revealed that illusory full-body ownership is elicited in similar magnitudes by synchronously stimulating different singular body parts. Adding to this existing literature, the current study found that illusory full-body ownership is also similarly elicited by the synchronous stimulation of one, two, or three body parts simultaneously. The only important factor was the synchronicity of the visuotactile stimulation, which was predictive of illusory full-body ownership ratings irrespective of the number of stimulated body parts. Consistent with the subjective results, there was no significant facilitatory effect of increasing the number of stimulated body parts on threat-evoked skin conductance responses or on full-body ownership illusion onset times (although see below for a detailed discussion on each of these measures). A previous study [41] compared illusory ownership during the rubber hand illusion and found that different combinations of multisensory information induced similar magnitudes of illusory ownership of the rubber hand. This observation led the authors to speculate that there was an ‘all-or-nothing’ character of the illusory feeling of ownership in the rubber hand illusion [41]. The notion of ‘all-or-nothingness’ also appears to fit with our findings here in the context of the full-body ownership illusion. Once enough multisensory evidence was accumulated by the synchronous stimulation of a single body part, the inference of ownership was established and remained constant with the stimulation of additional body parts. However, it should be pointed out that although body-part ownership for the rubber hand as a whole was reported as ‘all-or-nothing’ during the rubber hand illusion [41], the constituent parts of the rubber hand itself may reflect illusory ownership in a graded “spread”, for example, from stimulated to non-stimulated fingers [68]. Likewise, perhaps when body parts are perceived in the context of a greater whole, such as a full-body gestalt, they may be perceived at different magnitudes of illusory body-part ownership and independently of an ‘all-or-nothing’ full-body ownership illusion. Moreover, recent evidence from artificial neural networks and psychophysics experiments suggests that both graded and all-or-nothing multisensory percepts are computationally feasible [40].

Akin to illusory full-body ownership ratings (Q8), we found that the subjective ratings of illusory ownership for all of the body parts were significantly higher following synchronous stimulation than following asynchronous visuotactile stimulation, which adds to previous work demonstrating this effect for a subset of body parts [5,6]. The fact that body parts were perceived with significantly greater ownership when they were synchronously stimulated relative to when they were not stimulated was expected (see Introduction and [5]). However, the present study revealed several novel observations regarding the “spread of ownership” to non-stimulated body parts. Unlike previous studies [5,6], the “spread of ownership” was described not only for one stimulated body part to one or two other body parts but also for up to four other body parts symmetrically spaced over the entire body plan. Thus, our results provide more compelling evidence for a generalisation of ownership to all parts of the body. Second, we found that the increase in perceived ownership for non-simulated body parts did not increase as the number of synchronously stimulated body parts increased. Thus, the “spread of ownership” seemed to follow the pattern of the full-body ownership ratings (Q8) and be an “all-or-nothing” phenomenon, rather than linearly reflecting the total magnitude of visuotactile stimulation. Nevertheless, illusory ownership ratings for stimulated body parts were significantly predictive of those for non-stimulated body parts (albeit modestly, shared variance = 12.9%), which extends earlier findings of significant correlations between pairs of stimulated and non-stimulated body parts [5]. Thus, this result provides valuable corroborative evidence for the “spread” of illusory ownership between stimulated and non-stimulated body parts [5–7]. An equally exciting finding was the significant correlations and regressions between full-body ownership ratings (Q8) and the increase in illusory ownership for non-stimulated body parts when comparing synchronous with asynchronous conditions (shared variance = 15.3%). This result suggests that the rise in ownership for all non-stimulated parts is related to the full-body experience. We think this makes sense, since part and whole should be related, even if not identical constructs. Our analyses also suggest that the “spread” of body-part ownership might drive illusory full-body ownership (shared variance = 15.3%) to a greater extent than the elicitation of full-body ownership facilitating the “spread of ownership” across body parts (shared variance = 9%). However, as this may not be entirely clear from our data, it remains an interesting question for future studies.

While we observed significantly increased illusory ownership ratings for all five of the mannequin’s body parts following synchronous visuotactile stimulation, there were significant differences between different body parts. For example, illusory ownership for stimulated body pars was stronger than illusory ownership for non-stimulated body parts. However, there was also evidence to suggest that the mannequin’s right hemibody, which was often stimulated, was generally perceived with greater illusory ownership than the left hemibody, which was never stimulated (see also ‘Supporting Information –Left vs. right illusory ownership in asynchronous conditions’). We speculate that significant asymmetries in illusory body-part ownership in the current study may be explained by an attentional bias towards the right hemibody, as the present experiment did contain experimental conditions that applied stimulation to the right body parts and never to the left. We did not plan the study to examine possible lateralisation effects, so we did not record participants’ eye movements or use a fixation point, relying solely on participants’ adherence to the verbal instructions to attend to the entire body. However, bias in illusory ownership ratings towards the right hemibody was observed even when only the trunk, precisely along the body midline, was stimulated in condition 1S. This finding fits less well with the above explanation and indicates that there was either 1) covert attentional after-effects, which could be related to a cognitive expectation for right-sided limb stimulation given knowledge of the other experimental conditions or 2) a genuine lateralisation effect with weaker illusory ownership ratings for the left hemibody. Regardless of the underlying cause, the lateralisation effect seemed to be rather general since it was also observed when we analysed the data from asynchronous conditions. However, when analysing this difference using synchronous minus asynchronous ratings, we corroborated the importance of synchronously stimulating the right-sided limbs, as the difference was significant only for 2 and 3 body parts. To our knowledge, there are no investigations of potential lateralisation effects in the context of illusory full-body ownership perception. Future experimental studies should thoroughly examine this issue because we need to learn more about lateralisation in body ownership illusions and clarify how this relates to the literature on lateralisation of body representation [69,70], as well as neurological disorders of body representation [71,72].

In the current study, the objective test of the full-body ownership illusion using threat-evoked SCRs produced somewhat inconclusive results. Although we observed significantly stronger threat-evoked SCRs in the 1S condition, the trunk stimulation condition most similar to Petkova and Ehrsson’s [7] original illusion condition, compared to the 3A condition, the two planned comparisons 3S – 3A and 3S – 1S resulted in unexpected results that are not easy to explain. First, although the questionnaires indicated a very clear and significant difference in both full-body ownership ratings (Q8) and body-part ownership ratings for the threat-targeted body part, the left leg (Q7) for 3S – 3A, there was no significant difference in threat-evoked SCR. Second, the 1S condition produced stronger threat-evoked SCRs than did the 3S condition, although the questionnaires for these conditions indicated a similar subjective illusion for the whole body (Q8) and the threat-targeted body part (left leg; Q7). We had no reason to think that 1S could produce the strongest illusion (see introduction), and since previous studies have demonstrated successful SCRs using a non-stimulated body part, albeit a body part that was stimulated in another experimental condition [7], we did not anticipate any issues relating to the choice of the left leg as the threat-targeted body part. The threat-evoked SCR procedure has successfully been used in many full-body illusion studies contrasting synchronous and asynchronous conditions [7,16,17,25,29,73] and in numerous works on the rubber hand illusion [27,74–76]. The main difference between these studies and the present study is the choice of body part in which to present the knife theat. Here, the threat was presented to the left leg instead of the abdomen as in earlier full-body ownership illusion studies, which also means that the knife was deeper in the field of view than usual. We speculate that as the left leg was opposite to the right-sided stimulated body parts in the 3S condition, attention was attracted away from the threat stimulus and, therefore, reduced the arousal reaction. Future threat-evoked SCR experiments should re-evaluate this relationship using threats targeting the mannequin’s trunk, as well as further investigate these differences as potential lateralisation effects by examining threats targeting both the left and right limbs.

In addition to exploring whether increasing the number of synchronously stimulated body parts increases the subjective magnitude of the full-body ownership illusion, we investigated whether converging stimulation across multiple body parts could decrease the rate of illusion onset. Our analyses provided no evidence of significant reductions in the onset time of the full-body ownership illusion owing to whether one, two, or three body parts were stimulated. Thus, this result was consistent with the questionnaire results described above (for Q8) and collectively suggests that both the strength and the temporal onset of the full-body ownership experience do not depend on the number of stimulated body parts. However, our onset time estimates for Q8 were notably longer than those of previous studies [6,21], averaging 28 seconds for 1S, 25 seconds for 2S and 30 seconds for 3S. Therefore, it seems likely that the participants used more conservative decision criteria in the present study and, as the instructions explicitly emphasised that participants should press the button when they experienced illusory ownership for “the mannequin’s *whole* body”, indicated only when they indeed experienced a sense of ownership for each and every one of the mannequin’s body parts. Compared to the rubber hand illusion, the present onset times for full-body ownership are longer than some estimates for the classic setup (approximately 10 s) [11,77] but comparable for the set-up eliciting the illusion with synchronised finger movements, 23 seconds [64]. The slower full-body illusion onset times in the current study may also be explained by the fact that visuotactile stimulations were delivered less frequently than in previous studies [6,21], which allowed the experimenter to optimally deliver multiple stimulations simultaneously (see ‘Methods –Visuotactile stimulation’). Therefore, it could be that a critical component when interpreting illusion onset time is the number of visuotactile stimulations prior to illusion onset, rather than time per se. In the current study, the illusion was triggered after 4 to 5 tactile stimulations on average, which is not so different from [7], where the illusion was assumed to be elicited after 8 to 9 stimulations, although the frequency was faster in the latter case.

Returning to the notion of a “spread of ownership”, elsewhere in the literature and aside from subjective questionnaire ratings, [78] may present evidence of a widespread illusory ownership effect. The authors of [78] examined surface skin temperature (°C) during a third-person perspective virtual avatar illusion and compared experimental conditions involving spatially congruent (same body part stimulated) versus incongruent (not the same body part stimulated) visuotactile stimulation. Since the skin surface temperature (°C) of both the stimulated and non-stimulated body parts was reduced after congruent visuotactile stimulation, they concluded that a widespread temperature reduction is related to the experience of the full-body illusion [78]. However, it should be noted that the reported changes were in the range of 0.006–0.014°C, which the authors acknowledged as being very small. By way of criticism, the conclusion is based upon a temperature effect that was not consistent; reductions also occurred in one of the incongruent (control) conditions, and only one of the two comparisons between congruent and incongruent stimulations was statistically significant. There are also issues regarding the replicability and generalisability of skin temperature changes as an index of body illusions and body ownership [79,80]. While it is an intriguing possibility that illusory ownership of a false body or body part is related to a reduction in surface skin temperature, a precise hypothesis linking illusory ownership with the skin-cooling effect remains to be articulated.

### Limitations of the study

A limitation of the current study concerns the fact that the results are focused on the quantification of the subjective experience of body-part and full-body ownership via questionnaires with rating scales, which are vulnerable to task compliance, cognitive bias, and other post-perceptual factors. Furthermore, for our objective measurements using threat-evoked SCRs, it is possible that methodological differences between the current and former studies played a part in producing inconclusive results. In the present study, the SCR data were collected after the questionnaire experiment, which might mean that participants were less alert, and two threat events per condition were sampled at a fixed time point (at the end of each video). Earlier studies typically included three threat trials per condition presented at unexpected time points, conducted as shorter separate experiments. During the SCR experiment, participants were also doing the illusion onset task, and we do not know how conducting that task might have influenced the illusory experience compared to the initial questionnaire experiment. However, research on the rubber hand illusion suggests that the illusion is not affected by performing cognitive tasks [81]. However, in these previous SCR experiments, the participants did not undertake any prior task and simply relaxed [7]. This difference could also be worth pointing out, as the participant requirements in the questionnaire and SCR experiments were slightly different, meaning that it is slightly risky to directly compare detailed results across the two experiments.

## Conclusion

The current study supports a role for the generalisation of illusory ownership from part(s) to the whole during the full-body ownership illusion. However, illusory full-body ownership also represents a percept independent of illusory body-part ownership, as full-body ownership was not significantly enhanced by convergent multisensory stimulation of multiple body parts simultaneously. With novel manipulations of the full-body ownership illusion and the future application of modern neuroimaging techniques, we may gain exciting new insights into the neural mechanisms and computations responsible for the experience of both body-part and full-body ownership in the healthy adult human brain.

## Supporting information

S1 File (DOCX)

S1 Data (XLSX)
