# Supplementary material for: The contribution of stimulating multiple body parts simultaneously to the illusion of owning an entire artificial body
Source: PLoS One. 2021 Jan 25;16(1):e0233243. doi: 10.1371/journal.pone.0233243 (PMC7833142; doi:10.1371/journal.pone.0233243)
Supplement: S1 File — (DOCX) [file pone.0233243.s001.docx]

**Supporting Information**

| Table S1. Planned comparisons between the experimental items (Q1-Q8) and the control items (Q9, Q10) for synchronous conditions (1S, 2S, 3S) | | | | |
| --- | --- | --- | --- | --- |
| Questionnaire item | ***Z* score** | ***p* value** | ***p*_FDR_** | ***r*** |
| 1S | | | | |
| Q1 – Q9 | 5.550 | < .001 | .002 | .80 |
| Q2 – Q9 | 4.791 | < .001 | .002 | .69 |
| Q3 – Q9 | 3.790 | < .001 | .002 | .55 |
| Q4 – Q9 | 2.606 | .009 | .012 | .38 |
| Q5 – Q9 | 3.960 | < .001 | .002 | .57 |
| Q6 – Q9 | 2.403 | .016 | .0183 | .35 |
| Q7 – Q9 | 1.380 | .168 | .168 | .20 |
| Q8 – Q9 | 3.137 | .002 | .0032 | .45 |
|  |  |  |  |  |
| Q1 – Q10 | 5.673 | < .001 | .0013 | .82 |
| Q2 – Q10 | 5.277 | < .001 | .0013 | .76 |
| Q3 – Q10 | 4.319 | < .001 | .0013 | .62 |
| Q4 – Q10 | 3.135 | .002 | .0023 | .45 |
| Q5 – Q10 | 4.901 | < .001 | .0013 | .71 |
| Q6 – Q10 | 3.349 | < .001 | .0013 | .48 |
| Q7 – Q10 | 2.306 | .021 | .021 | .33 |
| Q8 – Q10 | 4.176 | < .001 | .0013 | .60 |
| 2S | | | | |
| Q1 – Q9 | 5.523 | < .001 | .0013 | .80 |
| Q2 – Q9 | 5.498 | < .001 | .0013 | .79 |
| Q3 – Q9 | 5.309 | < .001 | .0013 | .77 |
| Q4 – Q9 | 2.974 | < .001 | .0013 | .43 |
| Q5 – Q9 | 4.732 | < .001 | .0013 | .68 |
| Q6 – Q9 | 2.936 | .003 | .0034 | .42 |
| Q7 – Q9 | 1.639 | .101 | .101 | .24 |
| Q8 – Q9 | 4.108 | < .001 | .0013 | .59 |
|  |  |  |  |  |
| Q1 – Q10 | 5.536 | < .001 | .0016 | .80 |
| Q2 – Q10 | 5.508 | < .001 | .0016 | .79 |
| Q3 – Q10 | 5.200 | < .001 | .0016 | .75 |
| Q4 – Q10 | 2.497 | .013 | .0016 | .36 |
| Q5 – Q10 | 4.857 | < .001 | .0016 | .70 |
| Q6 – Q10 | 2.779 | .005 | .0067 | .40 |
| Q7 – Q10 | 1.589 | .112 | .122 | .23 |
| Q8 – Q10 | 4.090 | < .001 | .0016 | .59 |
| 3S | | | | |
| Q1 – Q9 | 5.544 | < .001 | .0013 | .80 |
| Q2 – Q9 | 5.282 | < .001 | .0013 | .76 |
| Q3 – Q9 | 4.862 | < .001 | .0013 | .70 |
| Q4 – Q9 | 2.164 | .03 | .0343 | .31 |
| Q5 – Q9 | 4.749 | < .001 | .0013 | .69 |
| Q6 – Q9 | 4.059 | < .001 | .0013 | .59 |
| Q7 – Q9 | 1.270 | .204 | .204 | .18 |
| Q8 – Q9 | 4.695 | < .001 | .0013 | .68 |
|  |  |  |  |  |
| Q1 – Q10 | 5.622 | < .001 | .0013 | .81 |
| Q2 – Q10 | 5.253 | < .001 | .0013 | .76 |
| Questionnaire item | ***Z* score** | ***p* value** | ***p*_FDR_** | ***r*** |
| Q3 – Q10 | 5.398 | < .001 | .0013 | .78 |
| Q4 – Q10 | 2.396 | .017 | .0194 | .35 |
| Q5 – Q10 | 5.102 | < .001 | .0013 | .74 |
| Q6 – Q10 | 4.436 | < .001 | .0013 | .64 |
| Q7 – Q10 | 1.473 | .141 | .141 | .21 |
| Q8 – Q10 | 4.772 | < .001 | .0013 | .69 |
| Table S1. Planned comparisons between the experimental items (Q1-Q8) and the control items (Q9, Q10) for synchronous conditions (1S, 2S, 3S). For these analyses, the ratings of the control items (Q9, Q10) were compared against the ratings of the experimental questionnaire items (Q1-Q8) using Wilcoxon’s signed ranks tests. The significant differences between these variables are a complementary validation that indicate that the participants’ responses to the experimental items reflect their experience of the illusion, as opposed to mere confabulation or task compliance effects.  Table S2. Planned comparisons between the control items (Q9 & Q10) for synchronous conditions (1S, 2S, 3S) | | | | |
| Questionnaire item | ***Z* score** | ***p* value** | ***p*_FDR_** | ***r*** |
| 1S | | | | |
| Q9 – Q10 | 1.182 | .237 | NA | .30 |
| 2S | | | | |
| Q9 – Q10 | 0.347 | .728 | NA | .05 |
| 3S | | | | |
| Q9 – Q10 | 0.612 | .541 | NA | .09 |

**Table S2. Planned comparisons between the control items (Q9 & Q10) for synchronous conditions (1S, 2S, 3S).** The lack of significant differences between the two control items shows that they similarly fulfil the role of control items in the questionnaire. NA = not applicable.

**Body Awareness Questionnaire**

**Body Awareness Questionnaire – Methods**

At the very end of the experiment, all 48 participants completed the 18-item Body Awareness Questionnaire (BAQ) by (1). The BAQ is a self-report scale for the measurement of individual differences in attentiveness to non-emotive everyday bodily processes, and its subscales address individuals’ attentiveness to 1) bodily responses or changes, 2) predictions of bodily reactions, 3) the sleep-wake cycle, and 4) the onset of illness. As a self-reported measure of individuals’ subjective interoception, it may capture the ‘interoceptive sensibility’ described by (2).

The logic behind the addition of this measure was to explore the potential relationship between individual differences in interoceptive sensibility and the magnitude of the full-body ownership illusion experienced by participants, reflecting an explorative attempt to account for some of the inter-individual variation in susceptibility to the full-body ownership illusion. As the trunk of the mannequin’s body remains still while the participants are breathing, we reasoned that this body part might have been related to the greatest discrepancy concerning accessible interoceptive signals. It is possible that individuals most attuned to their own internal bodily processes may show reduced illusory ownership for this specific body part. For example, (3) recently showed that illusory ownership of a virtual body could be induced using a breathing rhythm synchronized with participants’ actual breathing. Therefore, we also examined the correlation between participants’ self-reported BAQ scores and ownership ratings of the mannequin’s trunk.

Altogether, we used Spearman’s rank correlations to explore whether the BAQ scores were related to the subjective full-body ownership illusion ratings (Q8), illusory ownership ratings of the mannequin’s trunk (Q5), threat-evoked SCRs (µS) and full-body ownership illusion onset times (seconds). We ran these analyses on both the synchronous data and on the difference between synchronous and asynchronous data where possible (e.g., illusion onset times were not collected for asynchronous conditions). We also ran the correlations using averages of the separate BAQ subscales in place of the total BAQ scores.

**Body Awareness Questionnaire – Results**

BAQ scores were computed for each individual participant (N = 48) as the total of the ratings for each of the 18 items (mean = 74.77, SD = 13.20, SEM = 1.91). No consistent significant correlations were found between participants’ BAQ scores and illusory full-body ownership ratings (Q8), trunk ownership ratings (Q5) (including the difference between synchronous and asynchronous ratings), threat-evoked SCRs (µS) or full-body ownership illusion onset times (seconds). The correlation matrix for the BAQ, its subscales and illusory outcome variables are presented below (Supporting Information – Table S2).

**Table S3. Correlation matrix for the BAQ, its subscales and illusory outcome variables.**


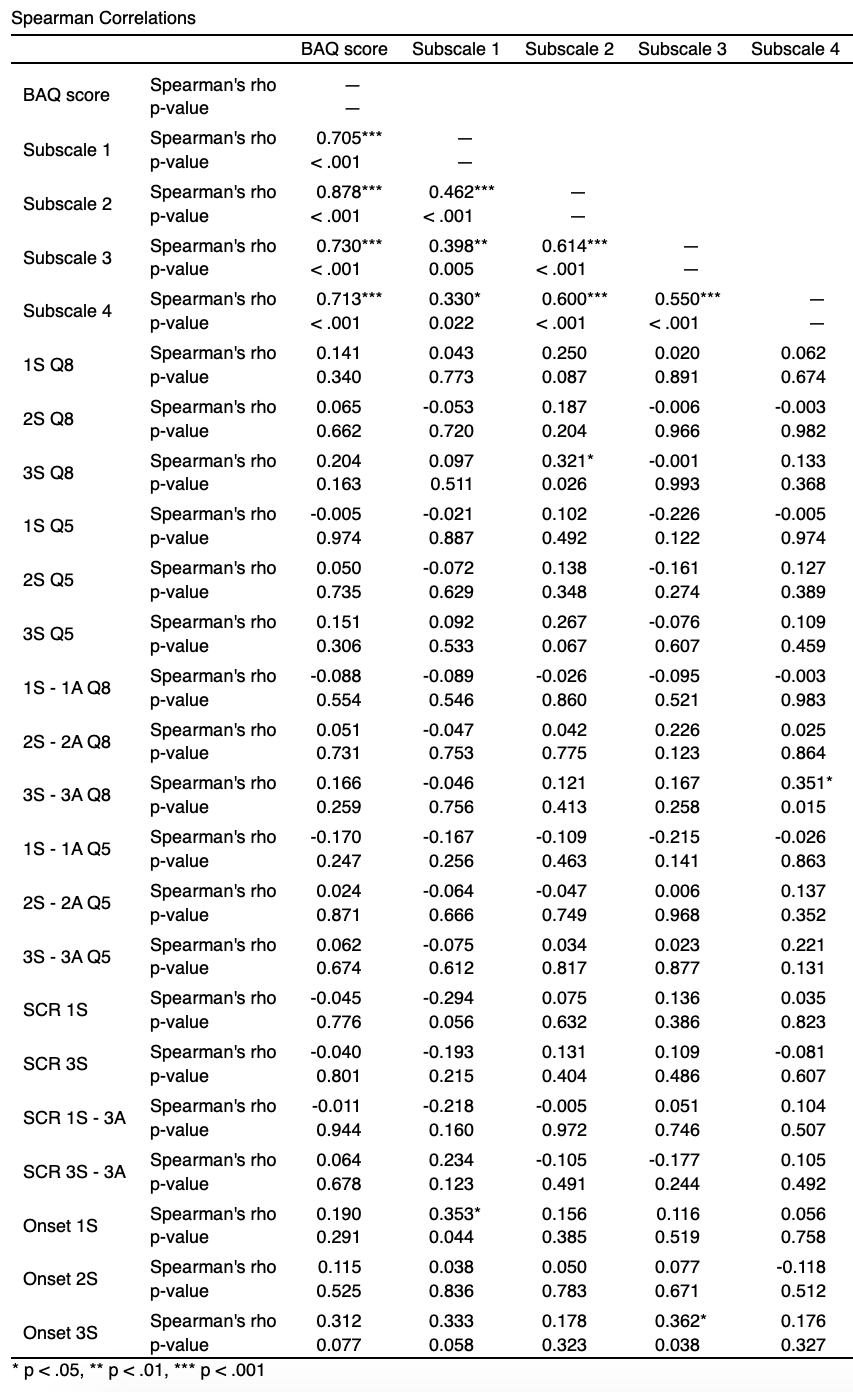


**Table S3. Correlation matrix for the BAQ, its subscales and illusory outcome variables.** Spearman’s correlations between the total BAQ scores and BAQ subscales (1: “note response or changes in body process”, 2: “predict body reaction”, 3: “sleep-wake cycle” and 4: “onset of illness”) and illusory full-body ownership ratings (Q8) for conditions 1S, 2S and 3S; illusory ownership ratings for the mannequin’s trunk (Q5) for conditions 1S, 2S and 3S; the difference between synchronous and asynchronous illusory full-body ownership ratings (Q8) (1S – 1A, 2S – 2A and 3S – 3A); the difference between synchronous and asynchronous illusory ownership ratings for the mannequin’s trunk (Q5) (1S – 1A, 2S – 2A and 3S – 3A) (all N = 48); threat-evoked SCRs (uS) for 3S, 1S, (3S – 3A) and (1S – 3A) (43 seconds) full-body ownership illusion times (33 seconds for full-1S, 2S illusion times (33). Spurious significant results are attributed to false positives since they are inconsistent across the experimental conditions. For example, 3S Q8 is significantly correlated with BAQ subscale 2 (*p* = .026). However, in 1S and 2S, neither questionnaire item is significantly correlated with BAQ subscale 2 (*p* = .204 and *p* = .087). The same is true for 3S – 3A Q8 and BAQ subscale 4 and onset time 3S and BAQ subscale 3. Due to their inconsistencies across the experimental conditions, these results likely reflect false positives, as there is a high number of correlations and all *p* values are uncorrected.

**Body Awareness Questionnaire – Discussion**

In failing to find any consistent significant correlations, we conclude that interoceptive sensibility does not appear to reflect individual variations of any relevance to the experience of a full-body ownership illusion induced by synchronous visuotactile stimulation. Other studies reporting a successful relationship between interoception and illusory ownership include those that induced the illusion of ownership over an avatar’s body using visual cues synchronised with interoceptive cues, such as participants’ heart rate (4). Our negative finding seems inconsistent with some studies suggesting a key role for interoceptive processing in the experience of body ownership (5,6) but is in line with other studies presenting results questioning this link (7). We reasoned that as the mannequin does not breathe, the subtle incongruences in the felt breathing movements of one’s own chest and the visual impressions of the mannequin’s stationary chest might provide visuo-interoceptive evidence against the full-body ownership illusion. Therefore, we speculated that participants with high BAQ scores may be more sensitive to this type of incongruence. However, our results provided no evidence for such a link. One possibility is that the BAQ is not sensitive enough to detect variations in interoceptive sensibility that contribute to the flexibility of full-body ownership during the perceptual illusion. However, it could also be that interoceptive sensitivity itself is less predictive of this variation in general. Other individual differences, for example, those directly related to the multisensory temporal binding window (8,9), may be more fruitful for future research examining the interindividual susceptibility towards body ownership illusions, as they have been for illusions in the audio-visual domain (10).

**Body Awareness Questionnaire - References**

1. Shields SA, Mallory ME, Simon A. The Body Awareness Questionnaire: Reliability and validity. J Pers Assess. 1989;53(4):802–15.

2. Garfinkel SN, Seth AK, Barrett AB, Suzuki K, Critchley HD. Knowing your own heart: Distinguishing interoceptive accuracy from interoceptive awareness. Biol Psychol [Internet]. 2015;104:65–74. Available from: http://dx.doi.org/10.1016/j.biopsycho.2014.11.004

3. Monti A, Porciello G, Tieri G, Aglioti SM. The “embreathment” illusion highlights the role of breathing in corporeal awareness. J Neurophysiol. 2020;123(1):420–7.

4. Suzuki K, Garfinkel SN, Critchley HD, Seth AK. Multisensory integration across exteroceptive and interoceptive domains modulates self-experience in the rubber-hand illusion. Neuropsychologia. 2013;51(13):2909–17.

5. Tsakiris M, Tajadura-Jiménez A, Costantini M. Just a heartbeat away from one’s body: Interoceptive sensitivity predicts malleability of body-representations. Proc R Soc B Biol Sci. 2011;278(1717):2470–6.

6. Crucianelli L, Metcalf NK, Fotopoulou A, Jenkinson PM. Bodily pleasure matters: Velocity of touch modulates body ownership during the rubber hand illusion. Front Psychol. 2013;4(OCT):1–7.

7. Crucianelli L, Krahé C, Jenkinson PM, Fotopoulou A (Katerina). Interoceptive ingredients of body ownership: Affective touch and cardiac awareness in the rubber hand illusion. Cortex. 2018;104:180–92.

8. Shimada S, Fukuda K, Hiraki K. Rubber hand illusion under delayed visual feedback. PLoS One. 2009;4(7):1–5.

9. Costantini M, Robinson J, Migliorati D, Donno B, Ferri F, Northoff G. Temporal limits on rubber hand illusion reflect individuals’ temporal resolution in multisensory perception. Cognition [Internet]. 2016;157:39–48. Available from: http://dx.doi.org/10.1016/j.cognition.2016.08.010

10. Stevenson RA, Zemtsov RK, Wallace MT. Individual differences in the multisensory temporal binding window predict susceptibility to audiovisual illusions. J Exp Psychol Hum Percept Perform. 2012 Dec;38(6):1517–29.

**Left vs. right illusory ownership for asynchronous conditions**

We averaged the left and right limb ownership ratings for asynchronous experimental conditions, which on average, generated a negative illusory ownership rating, and we also compared them using Wilcoxon’s signed ranks tests. For both 2A and 3A, there were significant differences between the left and right hemibodies, such that the right limbs were significantly less rejected than the left limbs, despite the former receiving an asynchronous visuotactile stimulation (2A: *Z* = 3.096, *p =* .002*, p_FDR_* = .003, *r* = .45; 3A: *Z* = 3.155, *p =* .002*, p_FDR_ = .*003, *r* = .46). This effect was not significant, however, for the 1A condition (*Z* = 1.863, *p = .*063*, p_FDR_* = .063, *r* = .27). Therefore, it is possible that the stimulation of the right limbs, albeit asynchronously, led to a reduction in participants’ confidence when denying the illusory experience of ownership for the right body parts, which could reflect cognitive bias effects. These results are presented below in Fig S1, which also shows, for reference, the results (all significant) of the difference between the averaged left versus averaged right limb ownership ratings under synchronous conditions (1S: *Z* = 2.530, *p =* .011*,* *p_FDR_* = .0132, *r* = .37; 2S: *Z* = 4.684, *p <* .001*,* *p_FDR_ =* .003, *r* = .68; 3S: *Z* = 4.955, *p <* .001*, p_FDR_* = .003, *r* = .72). Please refer to the main text for the results of the analyses conducted on synchronous minus asynchronous ratings, which showed that the asymmetrical effect remained significant for the 2 and 3 body-part conditions, suggesting that it is driven mainly by synchronous stimulation of the right-side body parts.

**
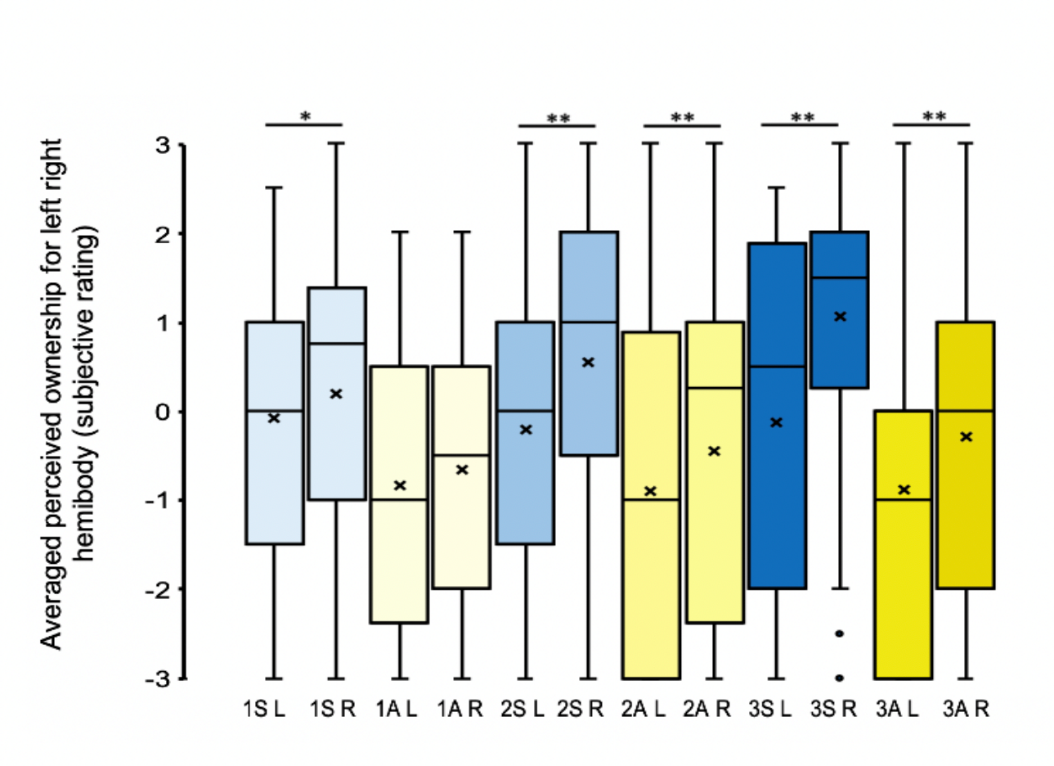
**

**Fig S1. Illusory ownership of the mannequin’s left versus right hemibody (averaged left and right limb ownership) across all experimental conditions. N = 48.** Perceived ownership of the mannequin’s left and right hemibody across both synchronous (blue) and asynchronous (yellow) visuotactile stimulation to one (lightest), two (intermediary) and three (darkest) body segments simultaneously. Apart from experimental condition 1A, the right body parts were experienced with significantly greater illusory ownership (synchronous conditions) or significantly reduced rejection (asynchronous conditions). The mean and median values are represented by the x and the straight line within the boxplot, respectively. Note: *** indicates significance at *p* < .001, ** indicates significance at *p* < .01, and * indicates significance at *p* < .05 after Benjamini-Hochberg FDR correction.
